# Increased HIV Testing Will Modestly Reduce HIV Incidence among Gay Men in NSW and Would Be Acceptable if HIV Testing Becomes Convenient

**DOI:** 10.1371/journal.pone.0055449

**Published:** 2013-02-15

**Authors:** Richard T. Gray, Garrett P. Prestage, Ian Down, Muhammad Haris Ghaus, Alexander Hoare, Jack Bradley, David P. Wilson

**Affiliations:** 1 The Kirby Institute, University of New South Wales, Sydney, Australia; 2 Australian Research Centre in Sex Health and Society, La Trobe University, Melbourne, Australia; University of Ottawa, Canada

## Abstract

**Objective:**

Determine the acceptability and epidemiological impact of increases in HIV testing in gay men in New South Wales (NSW), Australia– particularly pertinent when considering treatment as prevention and the need to reduce undiagnosed infections.

**Methods:**

We conducted an online survey and focus groups to assess whether increases in HIV testing would be acceptable to gay men in NSW. In parallel, we assessed the potential impact of increases in testing coverage and/or frequency using an individual-based model of HIV transmission.

**Results:**

If sexual practices and the rate of initiating HIV treatment are unchanged then increasing HIV testing reduces infections. Increasing testing frequency has the largest impact, with a 13.8% reduction in HIV infections over 10 years if the ∼55–75% of men who test at least once per year increased their testing frequency to four times per year. If testing levels decrease from current levels then we expect an increase in HIV infections with a sharply rising trend over time. Increasing HIV testing would be acceptable if testing was more convenient. However, only ∼25% of men surveyed were ‘very likely’ to increase their level of HIV testing. Men delayed or avoided testing due to the slowness in obtaining results and if they believed they had not put themselves at risk.

**Conclusions:**

An increase in HIV testing alone is unlikely to reduce HIV incidence substantially in NSW gay men– however, the relatively high testing levels need to continue to prevent an increase in HIV infections. In jurisdictions with lower levels of HIV testing, increases in testing coverage and frequency are likely to have a larger impact. Successful treatment as prevention interventions will require increases in testing rates; such increases would be acceptable to gay men in NSW but only if more convenient testing and rapid communication of results were available.

## Introduction

HIV testing is the foundation of HIV surveillance and health care for HIV-positive individuals. It is required to ensure infected individuals enter clinical care and receive appropriate treatment in a timely fashion. Testing is also fundamentally important for many HIV prevention initiatives. This is particularly true for populations of men who have sex with men (MSM) in high income settings as some risk reduction strategies rely on accurate knowledge of a potential partners’ HIV status. Also there has been a renewed emphasis on testing with the focus on treatment as prevention [1], [2]. Using treatment for prevention will only have the potential to work if infected people are diagnosed earlier through increased testing rates.

In Australia, HIV testing has provided knowledge of epidemiological trends through surveillance and informed the response to HIV and sexually transmissible infections (STIs). This response has been more effective than most comparable jurisdictions and has enabled the spread of HIV infection to be predominantly contained within populations of MSM, mostly gay men. It bears testament to the importance of both an evidence-based policy platform informed by HIV testing and an effective partnership between government, community, researchers and clinicians, as well as early and continuing mobilization within gay and other affected communities [3]. However, most Australian jurisdictions reported an increase in the number of HIV diagnoses over the last decade [4]. Similar trends have occurred in other countries in which HIV epidemics are predominantly driven by sex between men [5].

Within Australia, the state of New South Wales (NSW) has the highest population rate of HIV diagnosis but it has not observed increases in diagnosis rates in the last 10 years. This trend may not continue as there have been recent increases in unprotected anal intercourse with casual partners (UAIC) and in other STIs, including syphilis [4], [6]. These factors highlight the need to develop strategies that are likely to be both epidemiologically effective and socially acceptable.

Increases in HIV testing and screening, in particular, have numerous benefits, for the HIV-infected individual to receive care and management of infection and for public health surveillance systems to monitor epidemics. HIV testing can also reduce HIV transmission as diagnosed individuals may change their sexual behavior, by increasing their condom use or practicing risk reduction strategies, or receive antiretroviral treatment (ART) [7], [8]. A previous model investigated the expected relationship between testing rates and HIV incidence in Australian gay male populations [9] with the relative benefit of increased testing likely to decrease with higher testing coverage. Gay men in NSW already have a high rate of testing for HIV with approximately 55–75% of men self-reporting an annual test, based on age and level of risk [10]. However, self-reported estimates may be higher than actual testing rates [11]. Within Australia, testing for HIV is currently available through medical doctors and sexual health clinics, using a traditional venous blood sample for ELISA and Western Blot analysis. Also, until recently National HIV Testing Policy has mandated as standard of care both pre- and post-test ‘discussions’ (previously specified as counseling) [12] and test results have normally been obtained at a repeat visit to the clinic approximately one week later. Rapid testing and saliva-based testing options are not currently available in Australia for diagnostic purposes, although they have been supported in principle in recent changes to the National HIV Testing Policy [3].

We investigated the potential impact of interventions based on HIV testing on the HIV epidemic among gay men in NSW. This work used a mixed-method approach combining mathematical modeling to assess the potential epidemiological impact of various interventions with community-based social research to assess the acceptability of those same interventions within the target population. We examined the potential impact and community acceptability of increased testing coverage and frequency.

## Materials and Methods

Qualitative and quantitative data were collected on the beliefs and attitudes of NSW gay men concerning a range of interventions using focus groups and online surveys. We evaluated the potential impact of each type of intervention using a mathematical model of HIV transmission specifically developed to reflect this population.

### Ethics Statement

Ethics approval was obtained from the University of New South Wales and La Trobe University. The online survey was an anonymous survey and so written consent was not required. Participants were provided information about the study online and then indicated consent by proceeding to complete the survey. Focus group participants completed a written consent prior to their participation in these groups.

### Focus Groups and Online Survey

We recruited men for the online survey using advertisements on gay community websites. Focus group participants were recruited using the same online methods as well as through promotion at gay community events and venues. We conducted an online survey during May-June 2010. There were 309 men living in NSW who commenced the online survey of which 300 provided sufficiently complete information to be included in analysis. Among these 300 men, 233 were not HIV-positive (by self-report). Data reported here only includes men who were undiagnosed with HIV or did not know their serostatus.

Four focus groups were conducted in Sydney (two groups for HIV-positive men, and two for HIV-negative men) during May-June 2010. In all, 24 men took part in the groups and in terms of their demographics, those men could be considered broadly representative of sexually active urban gay men in Australia based on comparisons with demographic details from other studies we have conducted. We asked men to discuss how they perceive the risk of HIV transmission in general in their lives and what they would be prepared to do to avoid HIV transmission, based on a list of possible interventions.

Survey data were analyzed using SPSS ™ software to test for associations with having engaged in UAIC in the previous six months and with not having been tested for HIV within the previous year. Categorical variables were analyzed using Pearson’s chi-square test with Type I error of 5%. Focus group data were analyzed by a close reading of the transcripts by investigators who compared findings. This ensured that dominant themes in the transcripts were identified and coded as they emerged from the data through the technique of constant comparison [13].

### Modeling

We used an individual-based stochastic computer model to project the expected impact of HIV interventions on incidence. This model was specifically designed for gay men in NSW, Australia and is similar to other models we have developed and presented elsewhere [14], [15]. Here we provide a brief summary of the model focusing on HIV testing and its impact on behavior with an extensive and detailed description of all aspects of the model and our assumptions provided in Appendix S1 of the Supporting Information. A detailed listing and explanation for all the parameter values used in the model are given in Tables S1, S2, S3, and S4 in the Supporting Information.

The model was informed by extensive behavioral and epidemiological data available for gay men in NSW. It simulates the formation, sexual activity and breakup of regular, casual and group sexual partnerships in a population of 60,000 MSM who engage in anal intercourse, similar to the size of the identifiable MSM population in NSW, Australia [16]. Simulations of the formation, sexual activity and breakup of regular, casual and group sexual partnerships in the population of gay men are tracked over time. The model incorporates detailed sexual behavior and practices such as the practice of serosorting (where men select sexual partners who disclose having the same HIV serostatus) and strategic positioning (where during unprotected anal intercourse between serodiscordant partners the HIV-negative partner takes the insertive position to reduce the risk of transmission) [17]–[19]. Model variables describing infection and disease status of HIV, disease progression, treatment status, level of sexual activity, partnership availability, and current sexual partners of each individual are updated in daily time-steps. HIV transmission within the model population occurs between discordant partners during anal sex based on the characteristics associated with the sexual encounter. Diagnosed men initiate treatment at a rate that increases as their CD4 count decreases. Treatment initiation rates are set such that the majority of men do not begin treatment until their CD4 count falls below 350 cells/µl (further details in Appendix S1 of the Supporting Information).

The model consists of four main components: (1) Assigning population size and demographic characteristics (such as age profiles and circumcision rates); (2) Detailed sexual behavior associated with regular, casual and group partner acquisition and serosorting as well as behavior within partnerships such as frequency of sex, strategic positioning and condom use; (3) Tracking HIV disease progression and rates of testing and treatment; and (4) Modeling transmission between discordant partners during sex based on the characteristics associated with the sexual encounter.

Individuals are tested for HIV randomly each day with a probability per day that depends on the sexual behavior, age, and HIV status of each individual and the intervention simulated. These probabilities are defined such that the average testing levels among gay men matches to available population-level testing data. HIV-negative men are categorized into four sub-populations and given a testing probability calculated from the percentage tested for HIV each year (in the absence of an intervention) as reported in the Sydney Gay Community Periodic Survey (described in more detail in Table S4 in the Supporting Information) [16]. The four sub-populations are: (1) men younger than 30 who have low sexual activity (55% tested annually); men younger than 30 who have high sexual activity (65% tested annually); men older than 30 who have low sexual activity (65% tested annually); and men older than 30 who have high sexual activity (75% tested annually).

Surveys of Australian gay men show that for each of these population categories there is a proportion of the population who have never being tested for HIV [16], [20], [21]. This proportion is greater for younger men, as expected, given the dynamic nature of testing with men starting to test for HIV as they age or change their sexual behavior. However, there remain a small percentage of men aged greater than 30 years who have never tested for HIV (∼5%). The model categorizes men who have never tested for HIV as never testers. This categorization can change probabilistically when men turn 30 years old, so that never testers can begin HIV testing, to match the data from the behavioral surveys [16].

HIV testing can have an effect on incidence in the model through a number of features and assumptions. After diagnosis, men may reduce the number of sexual partners they have every 6 months. On average, we assume a 30% reduction in partner numbers post diagnosis. Individually this ranges from a 50% reduction to a 10% increase. We assume men diagnosed with HIV are more likely to disclose their serostatus to sexual partners. Men that disclose their positive serostatus are more likely to change their condom use and engage in serosorting and strategic positioning to reduce the risk of transmission to others. Although we did not investigate changes in the rate of ART initiation, testing can result in a greater number of treatment-eligible undiagnosed men beginning treatment. All details of these components as well as other data and assumptions for the model are presented in Appendix S1 of the Supporting Information.

The model was implemented using Matlab® R2010b with each simulation tracking the dynamic sexual network, HIV transmission, and disease progression of HIV-infected individuals. The model was calibrated to match the numbers of HIV diagnoses among gay men in NSW over the 1999 to 2009 period. For NSW, diagnoses attributed to MSM has varied from a maximum of 349 in 2003 to a minimum of 241 in 2009 as shown in Figure S2 of the Supporting Information. We ran each simulation for 50 years using the parameter values for the year 1996 to establish the network and dynamics of the model and ensure the HIV epidemiology in the model reached a steady state representative of available data for 1996. The resulting model population then represents the initial conditions for simulations from 1996 to 2010. From 1996 parameters were allowed to vary over time to match available data and the HIV transmission probabilities for insertive and receptive anal intercourse were calibrated (while remaining within empirical ranges; the resulting parameter values are shown in Table S3) so that the median of 50 simulations matched the available diagnoses data from 1999 to 2009. The 10 best-fitting model simulations (selected from the 50 simulations using a Pearson chi-squared test) were used to forecast epidemic trajectories from the end of 2010 for the next 10 years under various intervention scenarios (as described in Appendix S1 in the Supporting Information; the 10 best simulations are shown in Figure S1 and Figure S2).

To model the impact of interventions based on HIV testing, we simulated scenarios where the testing coverage and the frequency of testing were changed from their baseline values for particular prioritized populations (including the entire gay population). We also investigated scenarios that reduce the proportion of men who have never tested for HIV and scenarios where a large proportion of the prioritized population is tested over short time periods (referred to as synchronized or blitz testing). As gay men in NSW already test regularly for HIV, scenarios where HIV testing decreases in the future were also investigated to assess the importance of maintaining high testing levels.

## Results

### Acceptability Results from Online Survey

The online survey sample was broadly similar to what we have found in other surveys of non-positive gay men in Australia [22], [23]. Mean age was 38.6 years. Respondents were well-educated with 67.4% having received university-level education. Most identified as gay or homosexual (96.1%). Most men (92.3%) had been tested for HIV, with the majority (69.1%) indicating they had been tested in the previous year. Two-thirds (66.1%) reported having a regular male partner and 73.8% had had sex with any casual male partners in the previous six months. One-third (75 men, 32.2%) indicated that they had engaged in UAIC in the previous six months.

**Figure 1:**
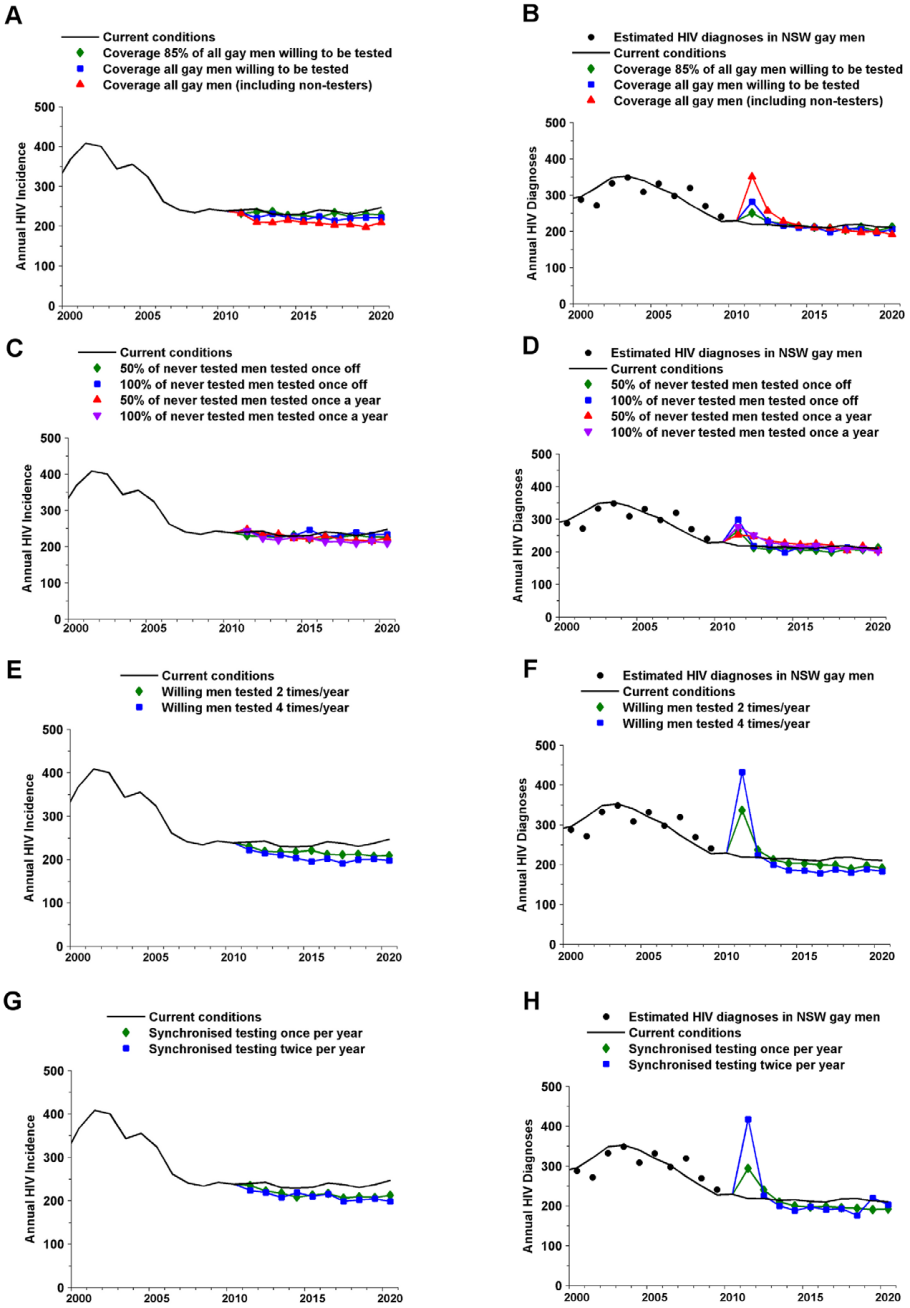
Likelihood of testing more frequently among non HIV positive men. (A) All non-positive respondents from online survey. (B) Non-positive men who engaged in UAIC. The data presented in the pie charts are available in Table S5 of the Supporting Information.

Men were asked to indicate their willingness to increase their testing frequency. Less than one in six men indicated any unwillingness to do so. Well over one-half of men indicated some willingness to increase their testing frequency, including one-third who said they were ‘very likely’ to do so (Figure 1). In these analyses, we have therefore regarded the men who indicated they were ‘very likely’ to increase their testing as being the best indicator of the men who are most likely to act on their hypothetical commitment. There was no difference in willingness to increase testing based on history of testing or of risk behavior (Table S5 in the Supporting Information). This was also true when analysis was conducted using only those men who provided a response to this question. The most common impediments to being tested and to testing more frequently were the requirement to return a second time to receive test results and the perception that they had not put themselves at risk of acquiring HIV (results shown in Table S6 in the Supporting Information). This varied according to respondents’ testing and condom use behavior. Men who had not been tested recently in the previous 12 months were more likely to report they had not engaged in risky behavior (48.5% of those not tested in past 12 months; p = 0.048). Men who reported no UAIC were more likely–compared to those who engaged in UAIC– to delay or not test because they had not changed partners (26.6% versus 12%; p = 0.008) or engaged in risky behavior (45.6% versus 26.7%; p = 0.004). However, men who had engaged in UAIC were more likely than those who did not to indicate that disclosure of possible positive status was a barrier to testing (44.0% versus 36.1%; p = 0.029). Only one in five men indicated that they never delayed or avoided being tested.

Survey respondents were asked what would encourage them to be tested more often, with most indicating that testing needed to be more convenient. Rapid saliva-based testing, non-clinic based testing facilities, self-testing and easier notification of test results were identified as ways to facilitate increased convenience (Table S7 in the Supporting Information). Among men who had not been tested recently, no single reason emerged ahead of others. They were less likely than were men who had been recently tested to be encouraged to test more if they could receive test results in twenty minutes (70.3% versus 54.4%; p = 0.016) or by phone (40.0% versus 26.5%; p = 0.034). Simplified procedures for receiving test results, such as by telephone or email, were viewed positively by only a minority of the sample overall, with a majority of the men who had engaged in UAIC indicated a preference for these methods for receiving their results.

When respondents were asked what kind of testing options they would prefer, saliva-based and rapid testing options were most commonly cited (Table 1). Current arrangements for receiving test results (i.e., returning in a few days) and establishing test sites at gay community commercial venues received the least support. Receiving test results via telephone or email elicited very mixed responses with many men preferring these options but considerably more men finding such methods ‘less preferable’. Men who had engaged in UAIC in the previous six months indicated more strongly that greater convenience would make it more likely that they would test (data not shown). It is also possible that these alternative options for testing could relieve them of the need to discuss their sexual behavior with their doctor.

**Table 1:** Testing option preferences for the 233 respondents of the online survey.

Testing Option		Less preferable (%)	About thesame (%)	More preferable(%)	Much more preferable (%)	No response (%)
Site	Free test site at gay venue	84 (36.0)	52 (22.3)	32 (13.7)	39 (16.7)	26 (11.2)
	Free test site at communityorganization	43 (18.4)	70 (30.0)	36 (15.5)	57 (24.5)	27 (11.6)
	Send own saliva or finger prickspecimen directly to laboratory	77 (33.1)	39 (16.7)	36 (15.5)	52 (22.3)	29 (12.4)
	Self-testing at home	59 (25.4)	30 (12.9)	48 (20.6)	71 (30.5)	25 (10.7)
Method	Saliva-based testing	12 (5.1)	49 (21.0)	36 (15.5)	111 (47.6)	25 (10.7)
	Finger prick testing	8 (3.4)	66 (28.3)	47 (20.2)	86 (36.9)	26 (11.2)
Delivery	Receive results by phone or SMS	90 (38.6)	20 (8.6)	33 (14.2)	61 (26.2)	29 (12.4)
	Receive results by email	91 (39.1)	18 (7.7)	33 (14.2)	60 (25.8)	31 (13.3)
	Receive test results in 20 minutes	7 (3.0)	16 (6.9)	48 (20.6)	133 (57.1)	29 (12.4)
	Return for test results thenext day	21 (9.0)	58 (24.9)	75 (32.2)	51 (21.9)	28 (12.0)
	Return for test results in a few days	37 (15.9)	97 (41.6)	41 (17.6)	26 (11.2)	32 (13.7)

The table shows the number of responses (with overall percentage in brackets) and preferences for various testing options.

### Focus Group Discussions

The main issues that emerged for men in the focus groups were: the need for more efficient and convenient testing procedures and facilities; the stress of having to wait for test results; and targeting the increased testing for men who were engaged in risky behavior. Many men also argued that testing was only relevant if someone was actually at risk of infection.

#### Convenience

Very commonly, men argued that HIV testing needs to be easier to access if they are to be asked to increase rates of testing. There were concerns raised by some men about confidentiality or appropriateness of settings aimed at making testing more convenient.

*If you’ve already got that obstacle ‘I’ve gotta go out of my way to be tested and now I’ve got to go out of my way twice a year to be tested’ then that’s kind of not gonna work. So it’s gotta be easier to get tested. The results have to come out faster. And the actual places you go to get tested have to be more friendly and more accommodating, and more welcoming.* (HIV-negative focus group)

#### Waiting for results

Many men referred to the need to return for a second visit to receive test results as a particular problem, either due to the inconvenience or the stress that often accompanies this waiting period.

*The… wait between testing and results is quite traumatic sometimes. Like when you go to the doctor and, you know, it’s another four days or something before the tests come back … Well it plays on my mind anyway, you know. Even, even though I probably 90 percent know I’ll be clear … if that process was somehow shortened or changed, then it would be better or less scary.* (HIV-negative focus group)

Reducing the amount of time involved in waiting for results through rapid testing was seen as one solution to this problem. Nonetheless, some men were less concerned by the time spent waiting for results and wanted to be assured that any other method of testing would deliver accurate results.

*If I had a choice between current method of testing and rapid testing, I guess whatever’s more likely to deliver an accurate result. The speed of it doesn’t really worry me.* (HIV-negative focus group)

Receiving results by email, SMS or by phone was discussed as a way of avoiding the need for a second visit to the doctor. Some men questioned the need to worry about the results at all unless they were positive.

#### Targeting risky behavior

Regardless of the availability of accessible testing options, some men argued that increased testing was only relevant for those men who had engaged in risky behavior:

*I don’t know because I am part of that 10 percent who don’t get tested and I don’t get tested because I don’t do anything or haven’t done anything, or had that accident for the reason to be tested. So going along to be tested is pointless for me, so I’d stay in that 10 percent no matter what anybody did or how anybody tried to influence me to go and get tested. It just wouldn’t mean anything to me.* (HIV-negative focus group*)*

### Modeling

**Figure 2:**
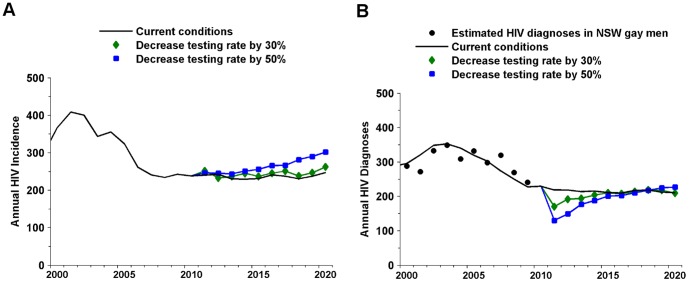
Mean change in HIV incidence and diagnoses due to increased testing. Change for: (A) and (B) increased testing coverage; (C) and (D) testing of men who have not been tested previously; (E) and (F) increased testing frequency; and (G) and (H) synchronized or blitz testing.

In the absence of testing interventions, the model suggests HIV incidence and diagnoses will reach an approximately steady level, with an average of 237 new infections and 218 new diagnoses per year for the following 10 years. The impact of increased HIV testing would be modest. Increasing the proportion of men who test for HIV each year is predicted to reduce annual HIV incidence to a lower level (Figure 2A). An 11.0% reduction in incidence over the next 10 years would be expected if all men, including those who never test for HIV, are tested once a year (range from a 0.8% increase to a 20.8% decrease across the 10 simulations (Table 2). Testing men who have never previously tested for HIV every year resulted in a 7.0% reduction in infections (ranging from a 6.2% increase to a 15.9% reduction – Table 2). Even if HIV testing frequency was to increase substantially, such that all men who currently test for HIV are tested four times per year, there would only be a moderate change in incidence, an estimated 13.8% reduction in HIV infections over the next 10 years (range from a 4.2% increase to a 20.6% decrease across the 10 simulations). There is little difference in projected effectiveness between synchronized/blitz testing and increasing testing frequency (Table 2). Interventions based on a combination of the different intervention types were determined to result in an additive effect. For all testing interventions, we expect an initial large spike in HIV diagnoses after their introduction (Figure 2). This means it may take at least two years before the true impact of these interventions will be detected by passive surveillance mechanisms that track new HIV diagnoses.

**Table 2:** Infections averted relative to the baseline case for screening interventions.

Intervention	Infections Averted(10 years)	Reductions in infectionsrelative to baseline
Increased testing coverage (Figures 2A and 2B)		
85% of gay men willing to be tested annually across all groups	69	2.6% (−6.4–12.6%)
100% of gay men willing to be tested annually across all groups	141.7	5.7% (−5.1–16.2%)
100% of gay men tested annually across all groups	266.7	11.0% (−0.8–20.8%)
Testing of men who have not been tested previously (Figures 2C and 2D)		
Test 50% of men who have never been tested – once off	−98.7	4.0% (−2.1–12.0%)
Test 100% of men who have never been tested – once off	26.9	0.8% (−19.1–11.7%)
Test 50% of men who have never been tested – annually	100.6	4.0% (−10.2–13.0%)
Test 100% of men who have never been tested – annually	173.7	7.0% (−6.2–15.9%)
Increased testing frequency (Figures 2E and 2F)		
Same testing coverage as current but increased frequency to twice per year	208.7	8.5% (−5.7–20.5%)
Same testing coverage as current but increased frequency to fourtimes per year	329	13.8% (−4.2–20.6%)
Synchronized or blitz testing (Figures 2G and 2H)		
Testing men (same coverage) in a one month period with currenttesting occurring in the background – once off	216.9	9.0% (−4.2–17.7%)
Testing men (same coverage) in a one month period with currenttesting occurring in the background – annually	267.5	11.1% (1.4–16.8%)

The second column shows the mean of the total number of infections averted during the 2010–2020 period for the 10 model simulations; negative numbers mean the number of infections increased in some simulations. The third column shows the mean and range for the percentage reduction in total infections.

**Figure 3:**
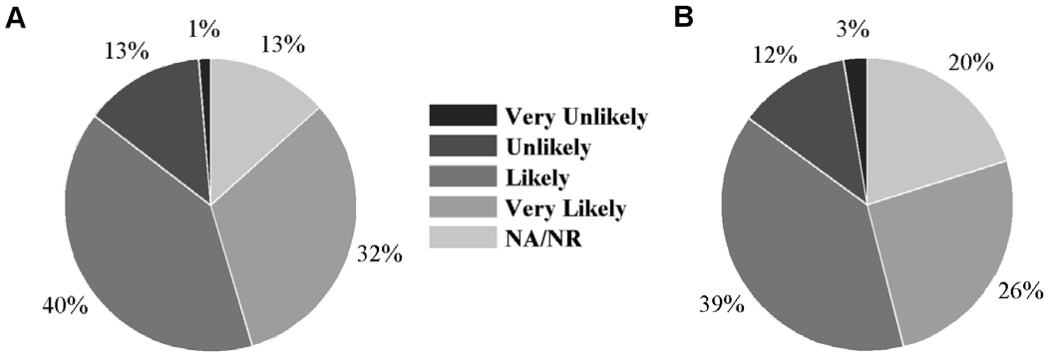
Mean change in incidence and diagnoses if testing rates decrease. Change in incidence (A) and diagnoses (B) if testing rates decrease by 30% and 50% relative to the current testing rate.

To investigate the impact and importance of HIV testing on HIV transmission in NSW gay men further we simulated the expected impact of reductions in testing coverage (Figure 3 and Table 3). If the rate of annual testing is decreased from its current level then it is likely that HIV incidence would increase in NSW gay men. A 50% reduction in the coverage of annual testing resulted in 54.6 additional infections in 2020 (a 23.6% increase relative to the baseline scenario) and 280.5 additional infections over the next 10 years (a 12.1% increase relative to the baseline scenario) (Table 3). This 50% reduction in testing results in an increasing trend over the next 10 years which is likely to continue beyond 2020 (Figure 3). This is in contrast to the increasing testing scenarios which result in incidence failing to a relatively constant level. It is important to note that this increase in incidence will not be reflected in HIV diagnoses which show a marked decrease before a gradual rise over the next decade to a similar level projected under current conditions (Figure 3B).

**Table 3:** Increase in HIV infections due to a decrease in testing rates.

Change in testing rate	Additional Infections(10 years)	Increase in infections relative to baseline
Decrease in annual testing coverage in men by 30%	79.4	3.6% (−4.9–17.7%)
Decrease in annual testing coverage in men by 50%	280.5	12.1% (4.0–25.5%)

The second column shows the mean of the total number of extra infections during the 2010 to 2020 period for the 10 model simulations. The third column shows the mean and range for the percentage reduction in total infections.

## Discussion

According to our mathematical modeling, interventions promoting increased coverage and frequency of HIV testing are likely to have modest direct impact on HIV epidemics among gay men in Australia. Testing is nonetheless central to the success of many other strategies particularly treatment as prevention. The effectiveness of increased testing is potentially reduced due to the already high coverage of testing currently occurring in NSW gay men which ranges between 55% and 70% each year [6], [10]. Our results replicate those from other modeling studies of Australian gay men [15], [24] and suggests the existence of a “saturation” of testing in the population. In contrast, if testing rates decline then infection rates may well increase. These results highlight the importance of maintaining the current high levels of testing. In addition these results also suggest that if there was a lower level of testing than that reported in the annual Gay Community Periodic Surveys [6], [10] (there is evidence of this in some jurisdictions [11]) then increases in testing will likely have a larger impact on HIV epidemics.

Increased HIV testing has broad acceptability to gay men at risk of infection. In general, non HIV-positive men were prepared to consider taking action to reduce the rate of infection among gay men, including increased testing. A number of challenges were raised that would need to be dealt with and there were concerns about the relative convenience and the possible burden of time and effort such increases in testing might require. This is especially important because as some gay men increasingly rely on non-condom-based risk reduction strategies, accurate and current knowledge of their own and their partners’ HIV status is essential to the relative success of such strategies.

This study used a mixed-methods approach bringing together social research and mathematical modeling to provide a description of both the likely effectiveness and feasibility of testing interventions aimed at reducing rates of HIV. Focused on gay men in NSW, Australia, this interdisciplinary approach has provided complementary information, which will help inform responses to HIV in that population and other similar populations in Australia and internationally. HIV testing has become particularly relevant recently with the focus on treatment as prevention [1], [2]. Although we did not investigate treatment as prevention interventions, such prevention strategies will only be successful if infected people are diagnosed earlier. This relies on increases in the coverage and frequency of HIV testing which will need to be acceptable to the relevant communities to be successful.

A limitation in the potential effectiveness of interventions promoting increased testing is the absence of alternative testing options, in particular the fact that rapid HIV testing remains unavailable within Australia. While current testing and treatment facilities are good, they are often inflexible or inconvenient and increased testing may be viewed men as an imposition unless adequate provisions are made to facilitate this. Most of the main barriers to testing identified by the men in this study, particularly the men who engaged in sexual risk behavior and their own preferred options for increased testing, concerned factors that could be most directly addressed through rapid, particularly saliva-based, testing options.

Applying these findings to other contexts needs to be considered with some caution. Our model population has similar characteristics to other populations of gay men and men who have sex with men in Australia and other high-income countries. Differences between NSW gay men and other jurisdictions would need to be considered when evaluating and applying our results. In addition, the limitations of our modeling need to be acknowledged. Our model does not incorporate the transmission of sexually transmitted infections (STIs), which could facilitate HIV transmission. A background level of STIs is effectively incorporated into the baseline HIV transmission probabilities used in the model but temporal variations in prevalence are not captured. While there has been a re-emergence of syphilis in NSW gay men over the last decade, since HIV testing does not directly affect STI transmission our results for the relative impact of increases in HIV testing are likely to be robust. Another limitation of our model is that it only crudely captures changes in testing rate and in the proportion of men who have never tested for HIV as men age. In terms of the social research, the survey consists of a large sample of homosexually active men in Australia but it was a volunteer convenience sample and may not be entirely representative of all gay or other homosexually active men. Similarly, four focus groups cannot capture sufficiently comprehensive responses to provide definitive conclusions.

### Conclusions

The acceptability research found that increasing testing rates would be acceptable to gay men in NSW if it is made more convenient. In particular, rapid HIV testing needs to be made available to Australian gay men, particularly those men at highest risk of infection and levels of testing should pertain to levels of risk for individual men. Mathematical modeling shows that increases in testing for HIV can lead to modest reductions in the number of new HIV infections. The current relatively high levels of testing are important to maintain as decreases in testing rates will result in an increase in HIV transmissions.

## Supporting Information

Figure S1 **The proportion of men in the model population who: are circumcised; have never been tested for HIV; have a HIV test each year; are HIV-positive and taking ART; and are on ART and have a detectable viral load compared to available data.**
(TIF)

Figure S2 **Annual HIV diagnoses from HIV in NSW model and the estimated number of HIV diagnoses in NSW gay men.**
(TIF)

Table S1 **Model parameters that describe the demographic characteristics of the MSM population in NSW.**
(DOCX)

Table S2 **Model parameters describing the sexual behavior characteristics of the MSM population in NSW.**
(DOCX)

Table S3 **Model parameters describing HIV biological characteristics of the MSM population in NSW.**
(DOCX)

Table S4 **Model parameters describing the clinical characteristics of the MSM population in NSW.**
(DOCX)

Table S5 **Likelihood of testing (more frequently) among non HIV-positive men (Survey 2). Number of responses recorded with percentage in brackets.**
(DOCX)

Table S6 **Reasons for delaying or not testing.** Number of responses recorded with percentage in brackets.
(DOCX)

Table S7 **What would encourage more men to test (more often)?.** Number of responses recorded with percentage in brackets.
(DOCX)

Appendix S1 **Technical description of the modeling methodology, parameterization, and calibration.**
(DOCX)
