# Supplementary figures and images for: Increased HIV Testing Will Modestly Reduce HIV Incidence among Gay Men in NSW and Would Be Acceptable if HIV Testing Becomes Convenient
Source: PLoS One. 2013 Feb 15;8(2):e55449. doi: 10.1371/journal.pone.0055449 (PMC3574096; doi:10.1371/journal.pone.0055449)

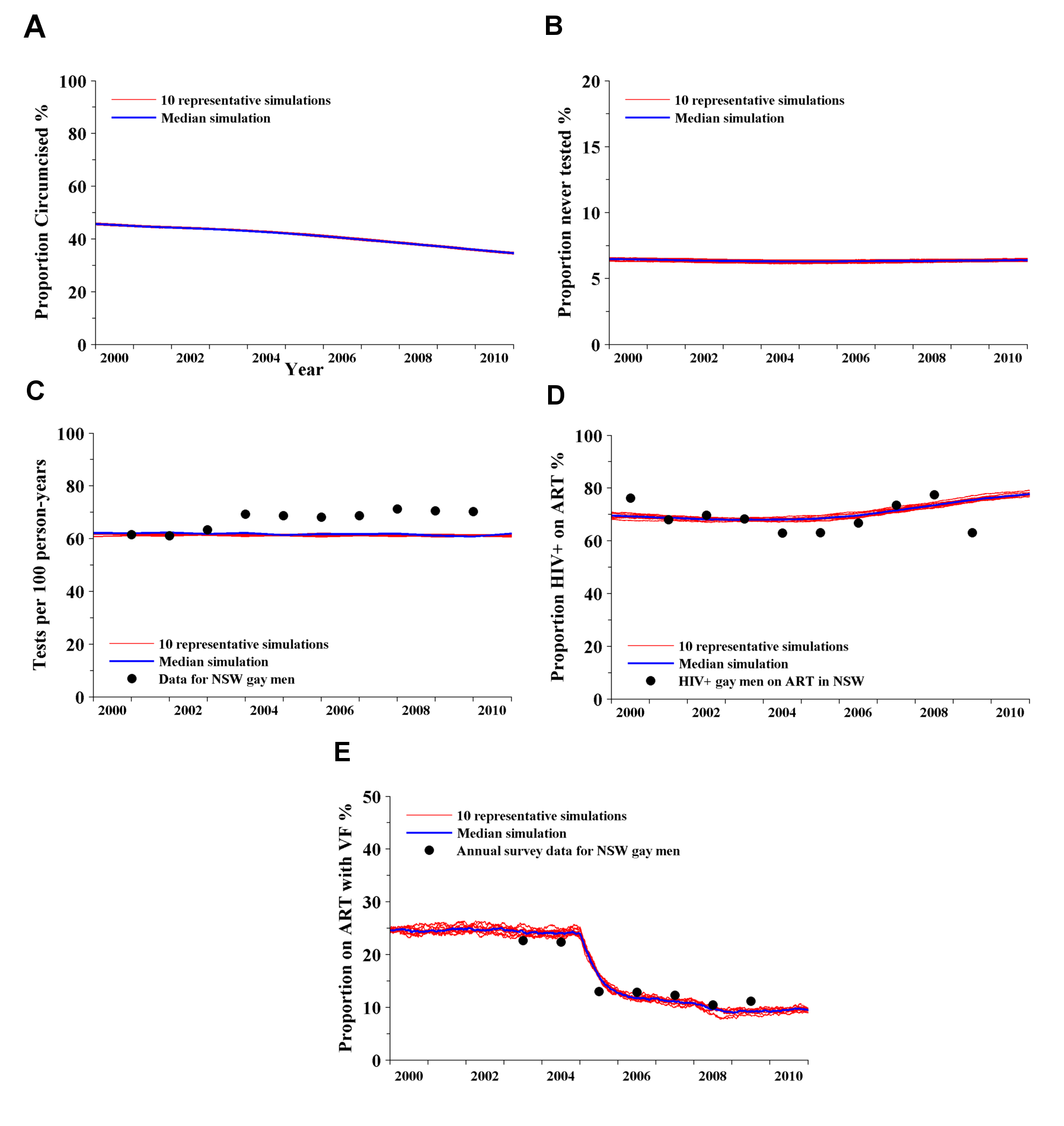

Supplement: Figure S1 — The proportion of men in the model population who: are circumcised; have never been tested for HIV; have a HIV test each year; are HIV-positive and taking ART; and are on ART and have a detectable viral load compared to available data. (TIF) [file pone.0055449.s001.tif]

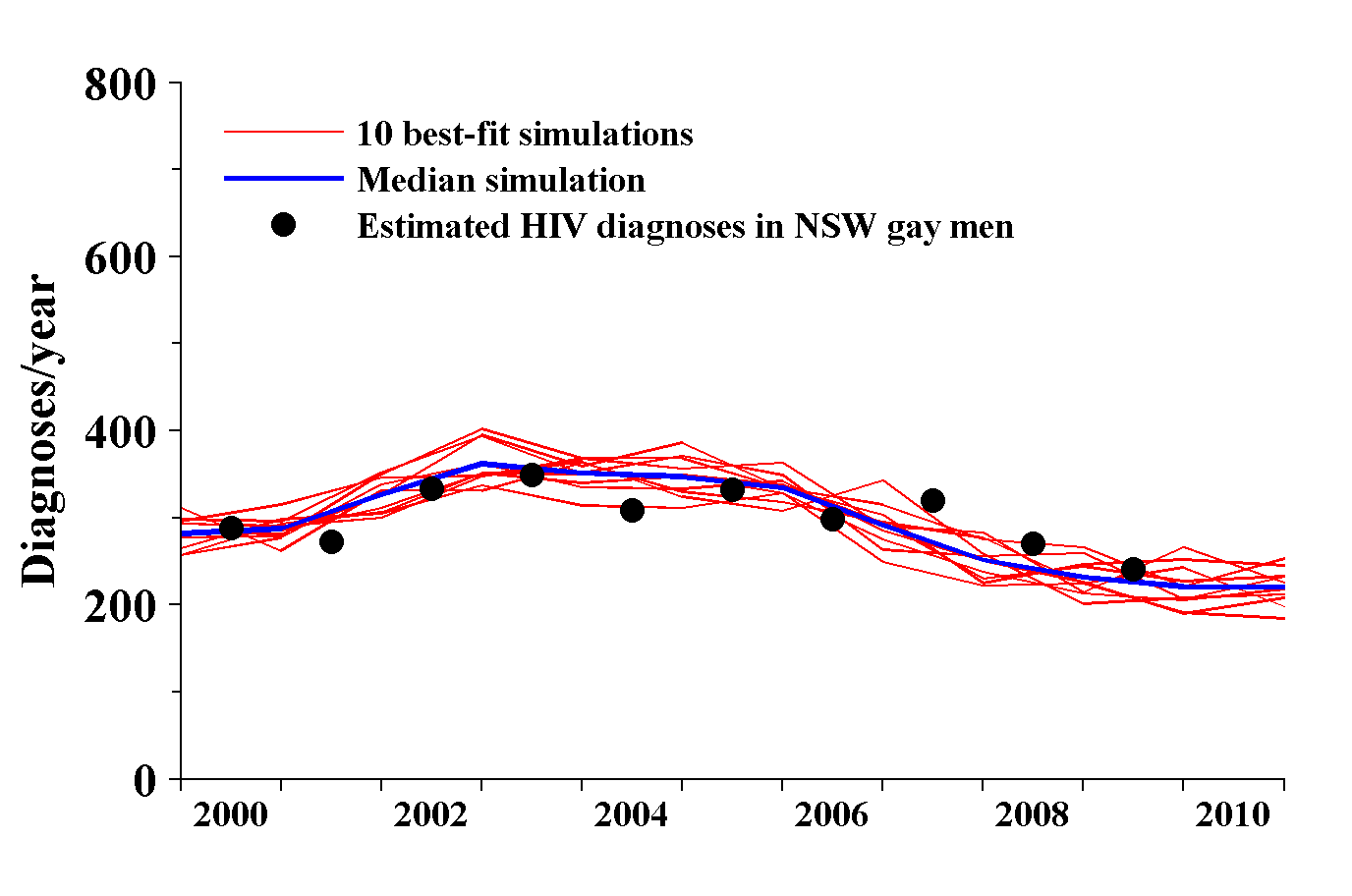

Supplement: Figure S2 — Annual HIV diagnoses from HIV in NSW model and the estimated number of HIV diagnoses in NSW gay men. (TIF) [file pone.0055449.s002.tif]
