# Supplementary material for: Increased HIV Testing Will Modestly Reduce HIV Incidence among Gay Men in NSW and Would Be Acceptable if HIV Testing Becomes Convenient
Source: PLoS One. 2013 Feb 15;8(2):e55449. doi: 10.1371/journal.pone.0055449 (PMC3574096; doi:10.1371/journal.pone.0055449)
Supplement: Table S1 — Model parameters that describe the demographic characteristics of the MSM population in NSW. (DOCX) [file pone.0055449.s003.docx]

**Table S1** - Model parameters that describe the demographic characteristics of the MSM population in NSW for the period from 1996 to 2010. Parameters are fixed for this period unless available data indicate there have been significant trends as described in the footnotes. The 2010 parameter values are used to represent current conditions.

| **Parameter Description** | | **Values** | **Reference** |
| --- | --- | --- | --- |
| **Demographic** | | | |
| Number of gay men in NSW | | 60,000 | [[2](#_ENREF_2)], a |
| Age of men entering the population | | 15-25 years | b |
| Age of men leaving the population due to old age | | 65-85 years |  |
| Proportion of gay men who are circumcised by age group in 1996 | < 25 years | 59.3% | [[13](#_ENREF_13)], c |
|  | 25-34 years | 69.5% |  |
|  | 35-44 years | 82.6% |  |
|  | > 45 years | 82.6% |  |
| Proportion of gay men entering the population after 1996 who are circumcised | | | d |
| * Model assumption based on discussions with expert stakeholders.  a: The model population is fixed at 60,000 sexually active gay men with young men entering the population as others age out or die.  b: These entry and exit ages are model assumptions based on the age range surveyed in the Sydney Gay Community Periodic Surveys (SGCPS) [[1](#_ENREF_1)]. In the 2007 survey, the median age was 34 years and the maximum age was 91 years.  c: This distribution is assumed for the gay male population in 1996. It is based on data from the HIM cohort which was recorded in 2001-2004 [[13](#_ENREF_13)]. In this cohort 50.5% of < 25 year olds, 59.3% of 25-34 year olds, 69.5% of 35-44 year olds, and 82.6% of > 45 year olds were circumcised. Since, individuals entering the population in 1996 will be aged between 22 and 32 by 2003 we use the 25-34 year old percentage for < 25 year olds in 1996 and similarly for the other age groups. In the model we assume there is no difference in circumcision distribution between HIV negative and positive individuals in 1996.  d: Males entering the population are aged between 15 and 25 years in the model and men entering the population between 1996 and 2009 could have been born between 1971 and 1995. There is limited data available for the proportion of male children in Australia who have been circumcised, however, over this time frame it is known that the circumcision rate has decreased dramatically, likely due to elective neonatal circumcision being no longer available in public hospitals in most states of Australia following changes in medical advice [[14](#_ENREF_14),[15](#_ENREF_15)]. In [[16](#_ENREF_16)] the number of fee-for-service neonatal circumcisions as a percentage of fee- for-service male births from 1979-80 to 1982-83 is published for each state in Australia and overall. Similar data has been published for the period 1994 to 1999 in Western Australia [[17](#_ENREF_17)]. These data are shown below:  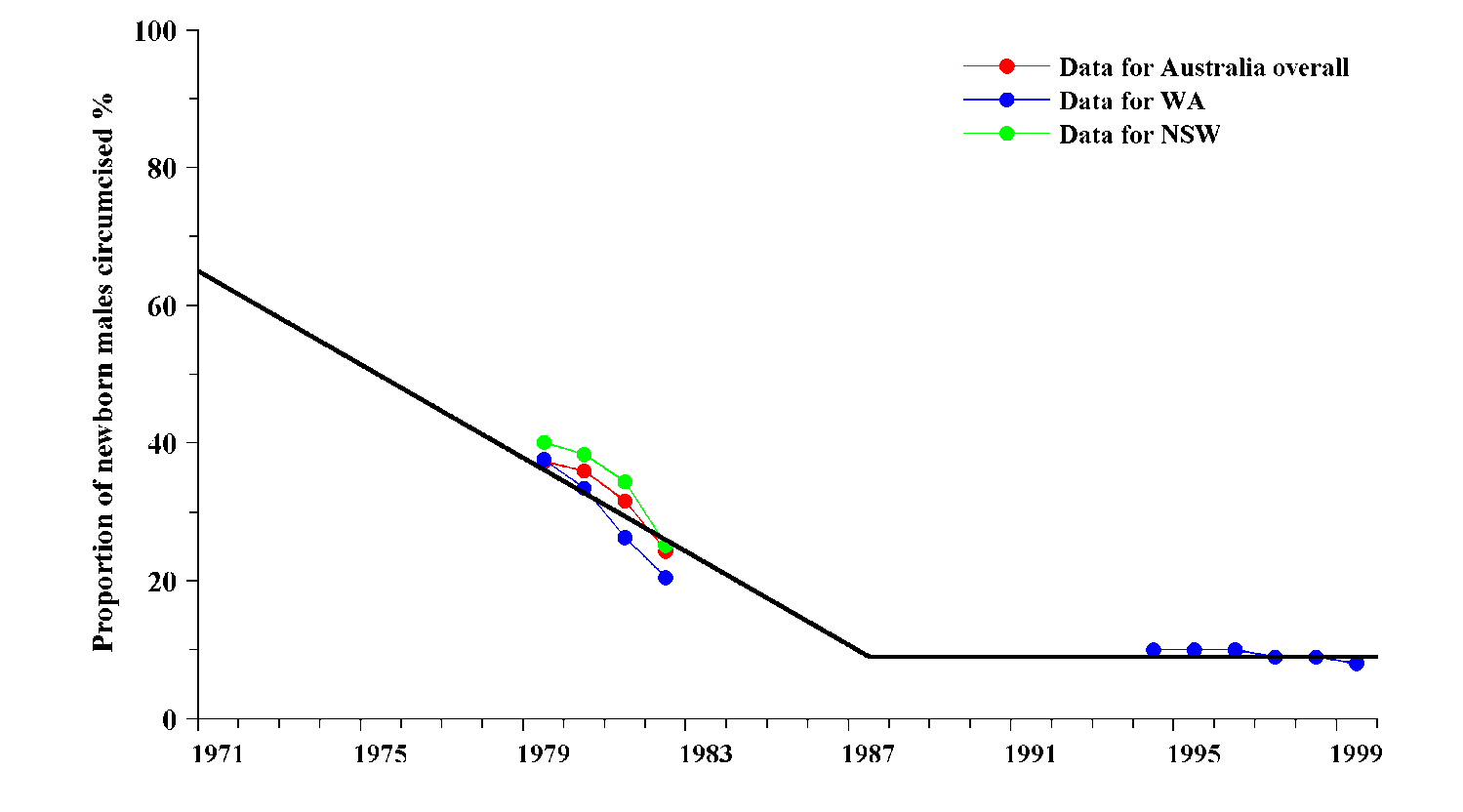  These data show there was a rapid decrease in circumcision rates between 1979 and 1983 which has levelled off to approximately a flat rate slightly less than 10% since 1994. For the model we fitted two straight lines to these data with a linear decrease from 65% in 1971 fitted to the 1979-83 data and a constant rate of 9% fitted to the 1994-99 data in WA. The 65% value for 1971 was chosen based on the percentage of men circumcised in the HIM cohort [[13](#_ENREF_13)] who are aged 25-34 in 2001-2004. The resulting percentage of men in the model population who circumcised is shown in Figure S1(a).  In the model the thick black line in the figure above gives the probability that a new man entering the population is circumcised. | | | |
