# Supplementary material for: Increased HIV Testing Will Modestly Reduce HIV Incidence among Gay Men in NSW and Would Be Acceptable if HIV Testing Becomes Convenient
Source: PLoS One. 2013 Feb 15;8(2):e55449. doi: 10.1371/journal.pone.0055449 (PMC3574096; doi:10.1371/journal.pone.0055449)
Supplement: Table S2 — Model parameters describing the sexual behavior characteristics of the MSM population in NSW. (DOCX) [file pone.0055449.s004.docx]

**Table S2** - Model parameters describing the sexual behavior characteristics of the MSM population in NSW from 1996 to 2010. Parameters are fixed for this period unless available data indicate there have been significant trends as described in the footnotes. The 2010 parameter values are used to represent current conditions.

| **Sexual Behavior** | | | | | | | | | |
| --- | --- | --- | --- | --- | --- | --- | --- | --- | --- |
| Distribution for the number of casual partners gay men have per 6 months (proportion of men in each category) | | 1-3 | | | 26% | | | | [[3](#_ENREF_3)] |
|  |  | 4-10 | | | 21% | | | |  |
|  |  | 11-20 | | | 16% | | | |  |
|  |  | 21-100 | | | 30% | | | |  |
|  |  | 101-120 | | | 7% | | | |  |
| Proportion of partnerships between men in the same 5 year age group | | | | | 30% | | | | [[8](#_ENREF_8)], e |
| Proportion of gay men who engage in group sex | | | | | 17% | | | | [[1](#_ENREF_1)], f |
| Multiplying factor for the change in number of sexual partners post diagnosis of HIV infection (this reflects a possible range from 50% decrease to 10% increase) | | | | | 0.5-1.1 | | | | [[5-7](#_ENREF_5),[18](#_ENREF_18)] |
| Percentage of gay men in a regular sexual partnership | | | | | ~38% | | | | [[1](#_ENREF_1)], g |
| Duration of regular partnerships | | | | | Mean 4 years; 95% range 36 days to 14.7 years | | | | [[2](#_ENREF_2)] |
| Average number of penile-anal acts with a regular partner per week | | | | | 2 (95% range 0-4) | | | | * |
| Average number of penile-anal acts per casual partner/encounter | | | | | 0.7 (range 0-1) | | | | [[1](#_ENREF_1)], h |
| Proportion of partnerships in which HIV serostatus is disclosed | | | | | | | | | i |
| Proportion of men who disclose their HIV infection status and serosort for  regular and casual partners who are seroconcordant | | | | | | | | | j |
| Proportion of sexual acts in which condoms are used for partnerships where the  HIV serostatus of one or both partners is unknown (equal to average condom use) | | | | | | | | | k |
| Proportion of sexual acts in which condoms are used for partnerships that are reported to be | | | | HIV concordant | | 0.2*average | | | l |
|  |  |  |  | HIV discordant | | (1-1.2)*average | | | m |
| Distribution of sexual position preferences | | | | | Insertive only | | | 33% | [[19](#_ENREF_19)] |
|  |  |  |  |  | Receptive only | | | 10% |  |
|  |  |  |  |  | Insertive and receptive | | | 57% |  |
| Sexual position matrix for HIV negative men who engage in UAI based on HIV serostatus of partner |  | | Receptive | | Receptive with withdrawal | | Insertive | | [[19-21](#_ENREF_19)], n |
|  | Partner reports negative | | 28% | | 17% | | 55% | |  |
|  | Partner status unknown | | 17% | | 22% | | 61% | |  |
|  | Partner reports positive | | 6% | | 18% | | 76% | |  |
| *Group sex parameters* | | | | | | | | | |
| Average number of group sex events per year for men who engage in group sex | | | | | 3.5 | | | | [[2](#_ENREF_2)] |
| Median number of men in each group sex event | | | | | Median 4.4 | | | | [[2](#_ENREF_2),[4](#_ENREF_4)] |
| Number of sexual partners in group sex event per person | | | | | Min: 1, Max: 10 | | | | * |
| * Model assumption based on discussions with expert stakeholders.  e: Other modelling work investigating the transmission of HIV in Australian gay men has estimated the level of assortativity of partnerships between gay men in terms of age [[8](#_ENREF_8)]. The results of this work can be interpreted to mean that 30% of a gay man’s partnerships are within their own age group (stratified in age bands of 5 years) with the remaining 70% of their partnerships randomly occurring with men in other age groups.  f: Recent studies of group sex among gay men in Australia suggest that most gay men have engaged in group sex at some time [[2](#_ENREF_2)] with 47% of HIV-negative gay men and 64% of HIV-positive gay men engaging in group sex in the previous 6 months [[1](#_ENREF_1)]. However, a large proportion of gay men only engage in group sex once off or very infrequently. The value here is the estimated value for the proportion of HIV negative gay men who engage in group sex regularly every year. We assume that only high activity gay men engage in group sex, however, for a person who engages in group sex, their total number of casual partnerships also includes their group sex partnerships. In our model the proportions of high activity men who engage in group sex is calibrated so that the overall proportion is equal to the values in the table.  g: In the model a casual partnership can develop into a regular partnership with a probability calibrated so that the proportion of gay men that have a regular partnership at any one time is 38%. This is slightly lower than the approximate 50% reported in the Gay Community Periodic Survey [[1](#_ENREF_1)].  h: Within a casual partnership men may engage in oral sex, anal sex, or both. We only consider anal sex for HIV transmission.  i: In the model gay men are designated to disclose their known HIV serostatus to regular, casual, and group partners. Data from the SGCPS [[1](#_ENREF_1)] gives the proportion of gay men who engage in unprotected anal intercourse (UAI) that always and never disclose their HIV serostatus. For regular partnerships a high proportion of men are assumed to always disclose their serostatus to their regular partners. Given the lack of data for this type of partnership it is assumed that the change in this proportion over time from 1996 to 2009 is constant at 90%.  For casual and group partnerships the proportion of men who engage in unprotected anal intercourse with casual partners (UAIC) and always disclose and who never disclose is reported in the SGCPS [[1](#_ENREF_1)] (this is assumed to be the same for group partnerships). Men are assigned to always disclose, sometimes disclose, or never disclose their serostatus in the model. The change in these proportions over time is shown in the following figure:  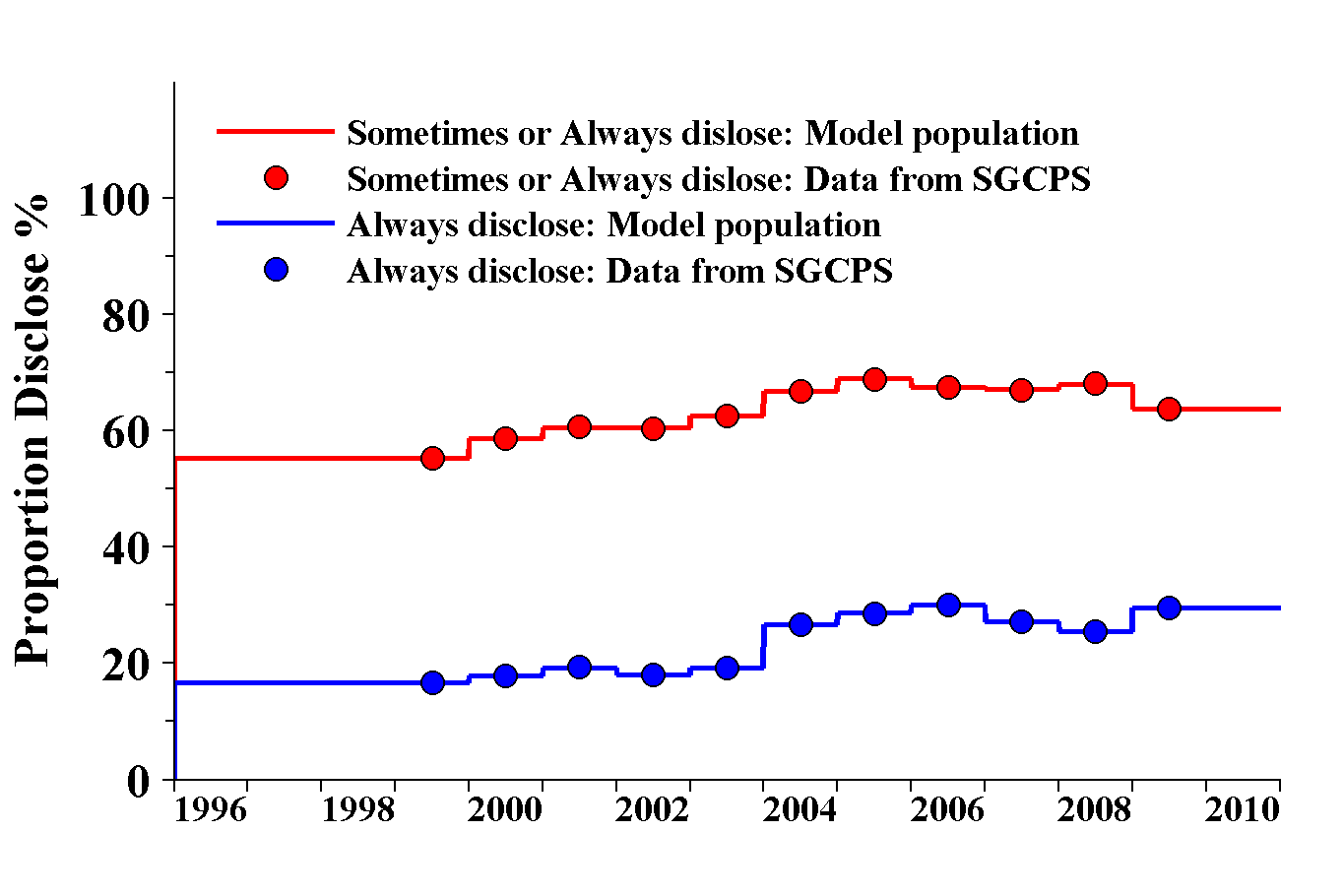  In the model, the proportion of men that always disclose and that never disclose is forced to match the exact data values from behavioral surveys, as shown in the figure above. The proportion of men assigned to sometimes disclose is equal to one minus the sum of the always and never disclose proportions. Men who sometimes disclose are assumed to disclose their HIV status to 20% of their casual/group partnerships. We assume the disclosure rates from 1996 to 1998 are the same as the 1999 levels which is the first year this type of data was recorded.  j: The practice of serosorting is hard to quantify using available data. The HIM and PH cohorts [[3](#_ENREF_3)] record the proportion of men who had UAIC with only seroconcordant casual partners from 2001 to 2006. These data are shown below:  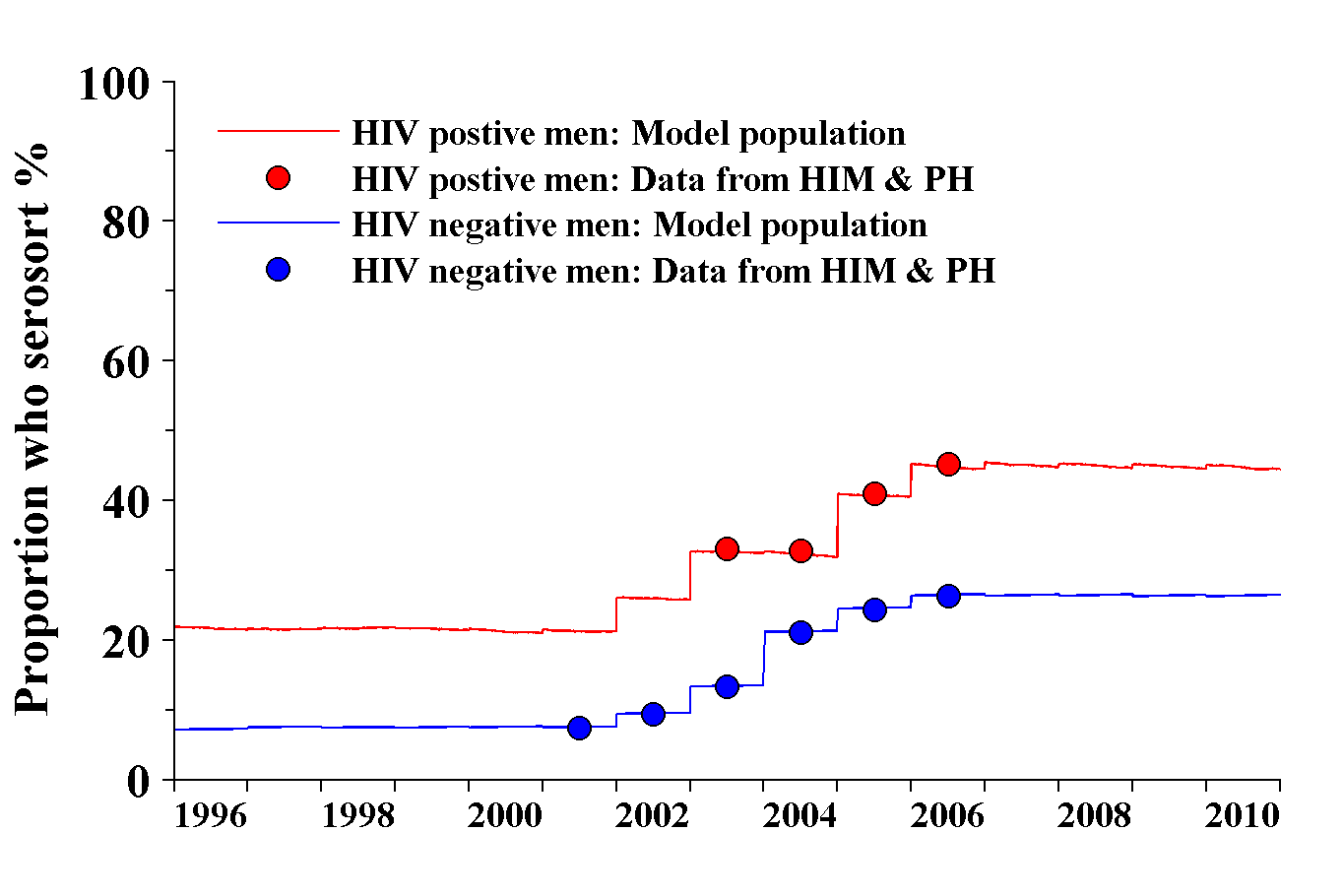  These data suggest that those who engage in UAIC serosort for partners with the same HIV serostatus with HIV positive men more likely to serosort for UAIC partners than HIV negative men and that serosorting for UAIC partners has been increasing over time since 2000. As HIV transmission is most likely to occur during UAI we assume the proportion of men who serosort is equal to the proportions recorded in the HIM and PH cohorts, as explained in the main text of the Supporting Information. Given that HIV transmission is most likely to occur during UAI we assume the proportion of men who serosort is equal to the proportions in the figure above to ensure that the proportion during UAIC matches these data. To determine the values of these proportions for the years when they were not recorded a linear relationship was initially fitted to these data to reflect the likely increase from 1996 to 2009. The level of serosorting prior to 2000 was set at a fixed level equal to the 1996 value. This 1996 value was calibrated so that the number of HIV diagnoses in 1996 matched the number of diagnoses in NSW during that year. After 2006 we assume the proportion of men who serosort is fixed at the 2006 value.    k: In the model condom usage is based on the whether a partnerships is discordant, concordant or unknown in terms of the disclosure of HIV serostatus. The SGCPS record the proportion of gay men who engage in UAI in casual partnerships. This is a reflection of condom use overall in the community and one minus this proportion gives the percentage of men who always use a condom during casual anal intercourse. The proportion of gay men who engaged in UAIC each year is shown below [[1](#_ENREF_1)]:  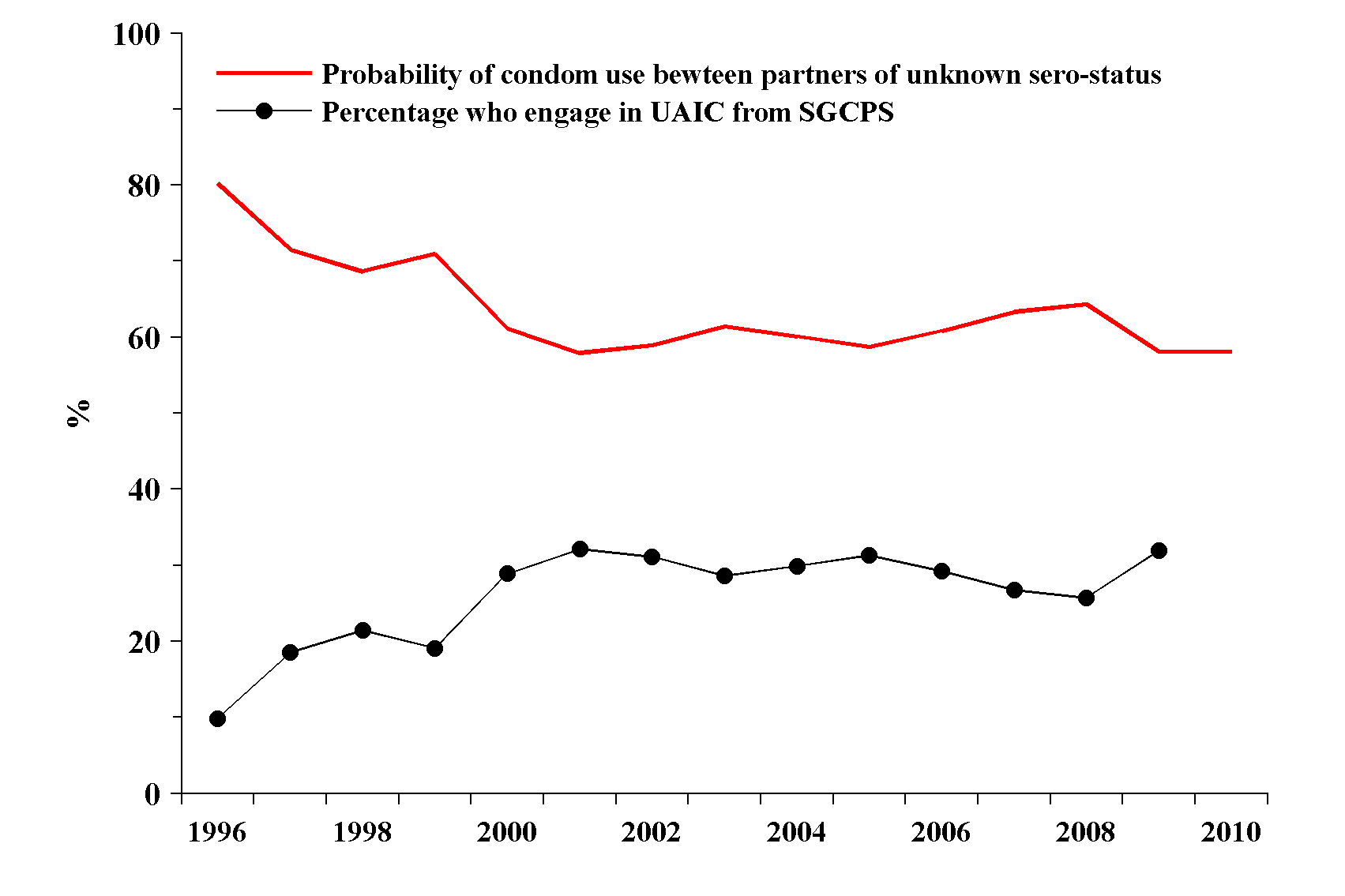  In the model the probability that a condom is used during AI in a partnership where the HIV serostatus of one or both partners is assumed to be 10% less than the proportion of gay men who always use a condom in casual partnerships.  l: The condom usage in partnerships that are thought to be HIV concordant is assumed to be very low. From the SGCPS 65% to 85% of gay men in regular seroconcordant HIV-positive partnerships and seroconcordant HIV-negative partnerships have engaged in UAI, respectively (for 2003 to 2009) [[1](#_ENREF_1)]. This means only 20-30% of men always use condoms in concordant regular partnerships over the multiple episodes of sexual intercourse. For the model we assume that condom usage rates for concordant partnerships is 20% of the rate for partnerships of unknown HIV serostatus. This varies from ~18% to 13%.  m: Condom usage in partnerships that are thought to be HIV discordant is assumed to be very high. From the SGCPS 45% to 39% of gay men in regular serdiscordant partnerships have engaged in UAI (for 2003 to 2009) implying 55 to 65% of men always use a condom in discordant regular partnerships [[1](#_ENREF_1)]. Given that this is over multiple sexual encounters, the probability of using a condom must be high. For the model we assume the probability that a condom is used per act in a discordant partnership is the minimum of 100% and 1.2 times the average probability (described in footnote k).  n: These values are based on a simple analysis of the sexual behaviour of men in the HIM cohort [[3](#_ENREF_3)]. In this cohort there are 1334 HIV negative men and the data presented gives the proportion of UAI acts these men had while in the cohort that were insertive, receptive with withdrawal, and receptive with ejaculation:  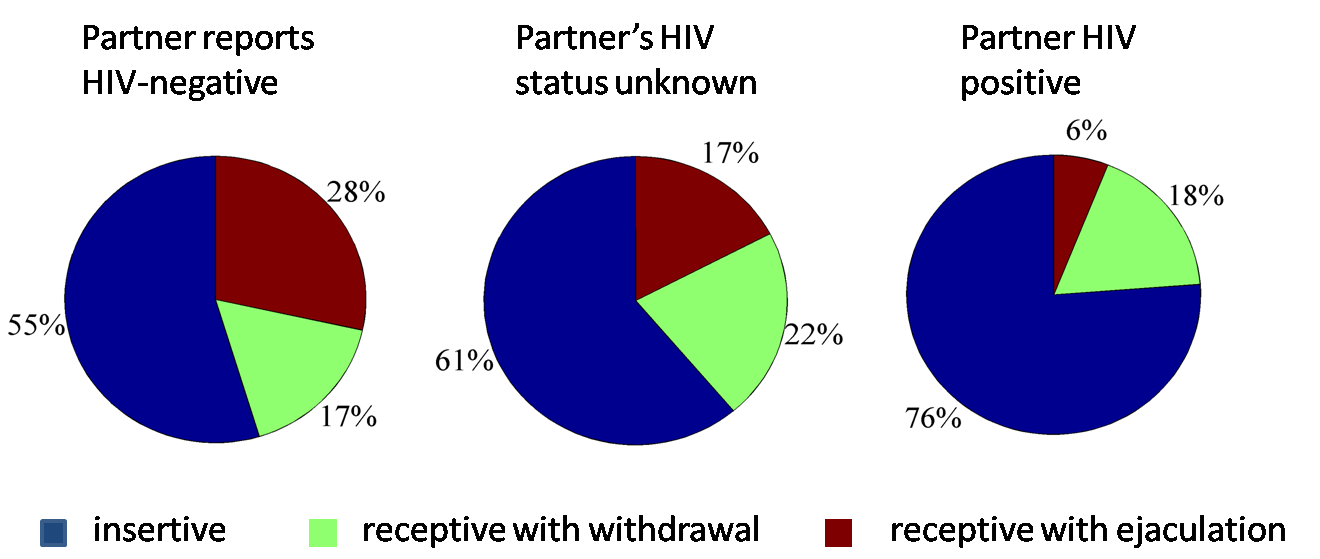 | | | | | | | | | |
