# Supplementary material for: Increased HIV Testing Will Modestly Reduce HIV Incidence among Gay Men in NSW and Would Be Acceptable if HIV Testing Becomes Convenient
Source: PLoS One. 2013 Feb 15;8(2):e55449. doi: 10.1371/journal.pone.0055449 (PMC3574096; doi:10.1371/journal.pone.0055449)
Supplement: Table S3 — Model parameters describing HIV biological characteristics of the MSM population in NSW. (DOCX) [file pone.0055449.s005.docx]

**Table S3** - Model parameters describing the HIV biological characteristics of the MSM population in NSW from 1996 to 2010. Parameters are fixed for this period unless available data indicate there have been significant trends as described in the footnotes. The 2010 parameter values are used to represent current conditions.

| **HIV Biological Parameters** | | | |
| --- | --- | --- | --- |
| *Disease Progression for men not on ART* | |  |  |
| Duration of Primary HIV stage | | 70-110 days | * |
| Time taken for HIV infected men to progress from: | CD4 count > 500 cells/μL to CD4 count 350-500 cells/μL | 1.79-4.42 years | [[22](#_ENREF_22)], o |
|  | CD4 count 350-500 cells/μL to CD4 count 200-350 cells/μL | 1.81-2.13 years |  |
|  | CD4 count 200-350 cells/μL to CD4 count < 200 cells/μL | 1.81-2.13 years |  |
| *Disease progression for men on ART* | | | |
| Time taken for HIV infected men on ART with undetectable viral load to progress from: | CD4 count < 200 cells/μL to CD4 count 200-350 cells/μL | 2.33-3.58 years | [[23](#_ENREF_23)], p |
|  | CD4 count 200-350 cells/μL to CD4 count 350-500 cells/μL | 0.90-3.42 years |  |
|  | CD4 count 350-500 cells/μL to CD4 count > 500 cells/μL | 1.07-7.28 years |  |
| *Death rates* | | | |
| Death rate per year for HIV infected men: | with CD4 count > 500 cells/μL and detectable viral load | 0.051% | [[24](#_ENREF_24)] |
|  | with CD4 count between 500 and 350 cells/μL and detectable viral load | 0.128% | [[24](#_ENREF_24)] |
|  | with CD4 count between 350 and 200 cells/μL and detectable viral load | 1% | [[24](#_ENREF_24),[25](#_ENREF_25)] |
|  | with CD4 count < 200 cells/μL and with detectable viral load | 5% | [[24](#_ENREF_24),[25](#_ENREF_25)], q |
|  | with CD4 count < 200 cells/μL and with undetectable viral load | 2% | r |
| *HIV transmission* | | | |
| Baseline per-contact probability of HIV transmission during UAI for insertive acts | Uncircumcised | Circumcised | [[11](#_ENREF_11)] |
|  | 0.42% | 0.074% |  |
| Baseline per-contact probability of HIV transmission during UAI for receptive acts | With withdrawal | With ejaculation | [[11](#_ENREF_11)] |
|  | 0.44% | 0.97% |  |
| Multiplicative change in HIV transmission probability from baseline  if infected partner: | is in primary stage of infection | 5.5 | [[9](#_ENREF_9),[26](#_ENREF_26)] |
|  | has AIDS | 5.5 | [[9](#_ENREF_9),[26](#_ENREF_26)] |
|  | is on effective ART | 0.08 | [[9](#_ENREF_9),[10](#_ENREF_10)] |
|  | is on ART but experiencing treatment failure | 1 | [[9](#_ENREF_9),[10](#_ENREF_10)] |
| * Model assumption based on discussions with expert stakeholders.  o: A summary of the relation between HIV-1 RNA concentration and decline in CD4 count from the prospective study by Mellors et al. [[22](#_ENREF_22)] is given below:   \| Plasma HIV-1 RNA  concentration (copies/mL) \| Mean decrease in CD4+ T cell  count per year (cells/µL) \| \| --- \| --- \| \| ≤ 500 \| 36.3 (30.4, 42.3) \| \| 501-3,000 \| 44.8 (39.1, 50.5) \| \| 3,001-10,000 \| 55.2 (50.7, 59.8) \| \| 10,001-30,000 \| 64.8 (59.6, 70.0) \| \| > 30,000 \| 76.5 (70.5, 82.9) \|   With these data, and assuming that the average viral load is ~104.87 copies per mL for people without treatment, the CD4+ T cell count decreases by an average of 76.5 (70.5, 82.9) every year.   - To progress through the >500 CD4 cell category, we assume that the average CD4 count is 800 cells/μL after the 2-month acute phase of HIV infection and then declines at the constant rate of 76.5 (70.5, 82.9) cells/μL each year. Then the average time to progress through this compartment is 2/12 + 300/(76.5 (70.5, 82.9)) years; that is 4.09 (3.79, 4.42) years. - To progress through the 350-500 and 200-350 CD4 cell categories, we assume an average loss of 150 CD4 cells. Then the average time to progress through this compartment is 150/(76.5 (70.5, 82.9)) years; that is 1.96 (1.81, 2.13) years.   p: Below is a summary of data from [[23](#_ENREF_23)] for changes in CD4 count over time among people who are on effective ART.   \| CD4 count at initiation of ART (cells per μL) \| Time since starting ART (years) \| Curent CD4 (cells per μL) means (95% CI) \| \| --- \| --- \| --- \| \| ≤ 200 \| < 1 \| 76 (53-99) \| \| 1-3 \| 69 (63-76) \| \| 3-5 \| 50 (36-69) \| \| > 5 \| 32 (18-46) \| \| 201-350 \| < 1 \| 129 (91-166) \| \| 1-3 \| 50 (25-74) \| \| 3-5 \| 47 (24-63) \| \| >5 \| 3 (2-44) \| \| > 350 \| < 1 \| 90 (37-144) \| \| 1-3 \| 50 (18-82) \| \| 3-5 \| 17 (-17-51) \| \| > 5 \| 2 (-12-54) \|   We used these data to estimate the average time to progress through our CD4 categories whilst on effective ART. For people with undetectable viral load:  • For CD4 count increases from 0 to 200 cells per µL, average increases of 76 (53-99) cells per µL can be expected during the first year and then 69 (63-76) cells per µL during the second and third years. Therefore, it can be expected to take 2.80 (2.33-3.58) years to progress through this category.  • For CD4 count increases from 200 to 350 cells per µL, we have a 150 CD4 count increase. In this interval, the CD4 count increases by 129 (91-166) cells per µL during the first year and then 50 (25-74) CD4 count during the second year. Therefore, it can be expected to take 1.42 (0.9-3.42) years to progress through this category.  • For CD4 count increases from 350 to 500 cells per µL, then we have a 150 CD4 count increase. In this interval, the CD4 count increases by 90 (37-144) cells per µL during the first year and then 50 (18-82) cells per µL during the second year. Therefore, it can be expected to take 2.20 (1.07-7.28) years to progress through this category.  q: HIV infected men with a CD4 count < 200 cells per µL that are not on treatment are assumed to have AIDS in the model and die within one year unless they start treatment. If they begin treatment but experience treatment failure their death rate is the percentage given in the table.  r: The death rates for men on ART are determined by their CD4 count (which increases over time if they are on effective ART) and is assumed to be the same as for men with detectable viral load. | | | |
