# Supplementary material for: Increased HIV Testing Will Modestly Reduce HIV Incidence among Gay Men in NSW and Would Be Acceptable if HIV Testing Becomes Convenient
Source: PLoS One. 2013 Feb 15;8(2):e55449. doi: 10.1371/journal.pone.0055449 (PMC3574096; doi:10.1371/journal.pone.0055449)
Supplement: Table S4 — Model parameters describing the clinical characteristics of the MSM population in NSW. (DOCX) [file pone.0055449.s006.docx]

**Table S4** - Model parameters describing the clinical characteristics of the MSM population in NSW from 1996 to 2010. Parameters are fixed for this period unless available data indicate there have been significant trends as described in the footnotes. The 2010 parameter values are used to represent current conditions.

| **Clinical Parameters** | | | |
| --- | --- | --- | --- |
| Per-act reduction in HIV transmission by condoms | | 95% | *, s |
| Percentage of the gay population that have never tested for HIV | < 30 years; Low sexual activity | 15% | [[1](#_ENREF_1)], t |
|  | < 30 years; High sexual activity | 8% |  |
|  | >= 30 years; Low sexual activity | 6% |  |
|  | >= 30 years; High sexual activity | 5% |  |
| Percentage of undiagnosed gay men who test for HIV each year in the absence of a specific intervention | < 30 years; Low sexual activity | 55% | [[1](#_ENREF_1)], u |
|  | < 30 years; High sexual activity | 65% |  |
|  | >= 30 years; Low sexual activity | 65% |  |
|  | >= 30 years; High sexual activity | 75% |  |
| Sensitivity of HIV test | | | v |
| *HIV Treatment* | | | |
| Proportion of HIV+ with a CD4 count > 500 cells/μl who go on treatment each year  from 1996 to 2008 | | 0.5% | *, w |
| Proportion of HIV+ with a CD4 count between 500 and 350 cells/μl who go on  treatment each year from 1996 to 2008 | | 20% |  |
| Proportion of HIV+ with a CD4 count between 350 and 200 cells/μl who go on treatment each year from 1996 to 2008 | | 40% |  |
| Proportion of HIV+ with a CD4 count < 200 cells/μl who go on treatment each year from 1996 to 2008 | | 60% |  |
| *Treatment failure* | | | |
| Proportion of men that start ART who experience treatment failure at initiation | | | x |
| Proportion of men on ART who experience viral rebound each year | | 5% | [[27](#_ENREF_27)] |
| *Viral load testing* | | | |
| Time period between viral load tests for HIV positive men on ART to detect if treatment failure has occurred | | 3 months-1 year | * |
| * Model assumption based on discussions with expert stakeholders.  s: This value is assumed based on estimates of condom effectiveness and data on condom breakage and slippage during anal intercourse between MSM. A systematic review of condom effectiveness in partnerships found consistent condom use reduced HIV incidence by 80%—where consistent use is using a condom for all acts of penetrative vaginal intercourse [[28](#_ENREF_28)]. The per-act effectiveness has to be greater than this 80% estimate for consistent condom use. A recent study reported breakage, slippage, and partial use errors occur frequently in MSM partnerships in the US [[29](#_ENREF_29)]—and we would expect similar results for Australian MSM. In the study, out of 1650 condoms used, breakage and slippage occurred in 3.4% of acts and partial use errors (delayed use or early removal) occurred in 11.2% of acts (14.6% overall). The effect of these problems with condoms on transmission is unknown—breakage, slippage and removal before ejaculation could result in no protection but delayed use might only reduce protection slightly. These results reflect previously published results where 1% to 8% of protected acts had condom breakage or slippage during heterosexual intercourse (depending on experience) [[30](#_ENREF_30)]. Based on this data for condom failure and the effectiveness estimate for consistent condom use we assumed a 95% reduction in per-act HIV transmission by condoms.  t: From the SGCPS there is a proportion of the population that has never tested for HIV. From 1996 to 2009 the rates for highly sexually active (HSA) (defined to be those with more than 10 casual partners every 6 months) and low sexual activity men (LSA) (defined to those who have less than 10 sexual partners every 6 months) gay men who are younger and older than 30 years of age are shown below [[1](#_ENREF_1)]:  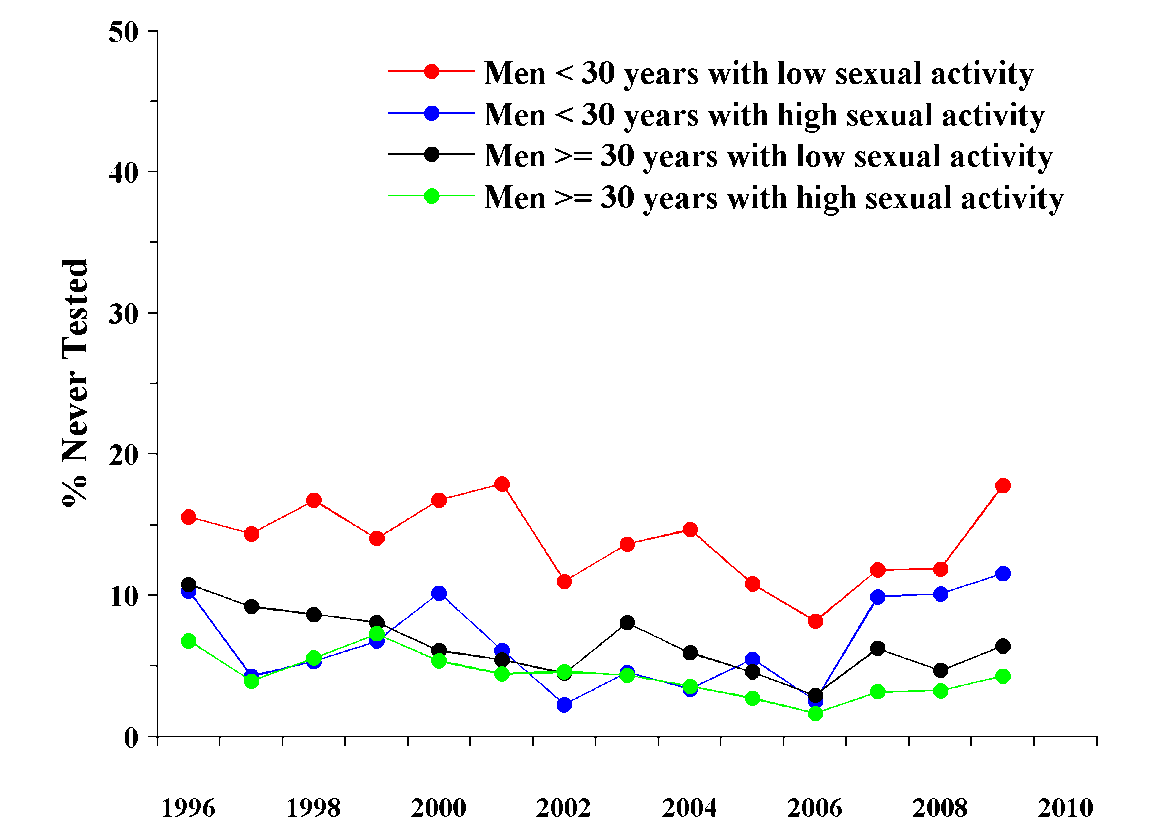  These data show there is a relatively constant level of men who have never tested for HIV with a larger proportion of younger LSA gay men having never tested for HIV (~15%) compared to the rest of the population (5-10%). In the model we assign proportions of men in each population category to never test for HIV with a fixed probability. When younger men who have never tested turn 30 there is a probability that they will become available to be tested for HIV to match the proportions for older men. The overall proportion of men who have never been tested for HIV in the model population is shown in Figure S1(b).  u: The probability that a HIV negative individual is tested for HIV in a given year is based on the proportion of gay men who tested for HIV in the previous 12 months in the SGCPS. For men who have previously tested for HIV the percentage who tested in the previous 12 months from 1996 to 2009 is shown below [[1](#_ENREF_1)]:  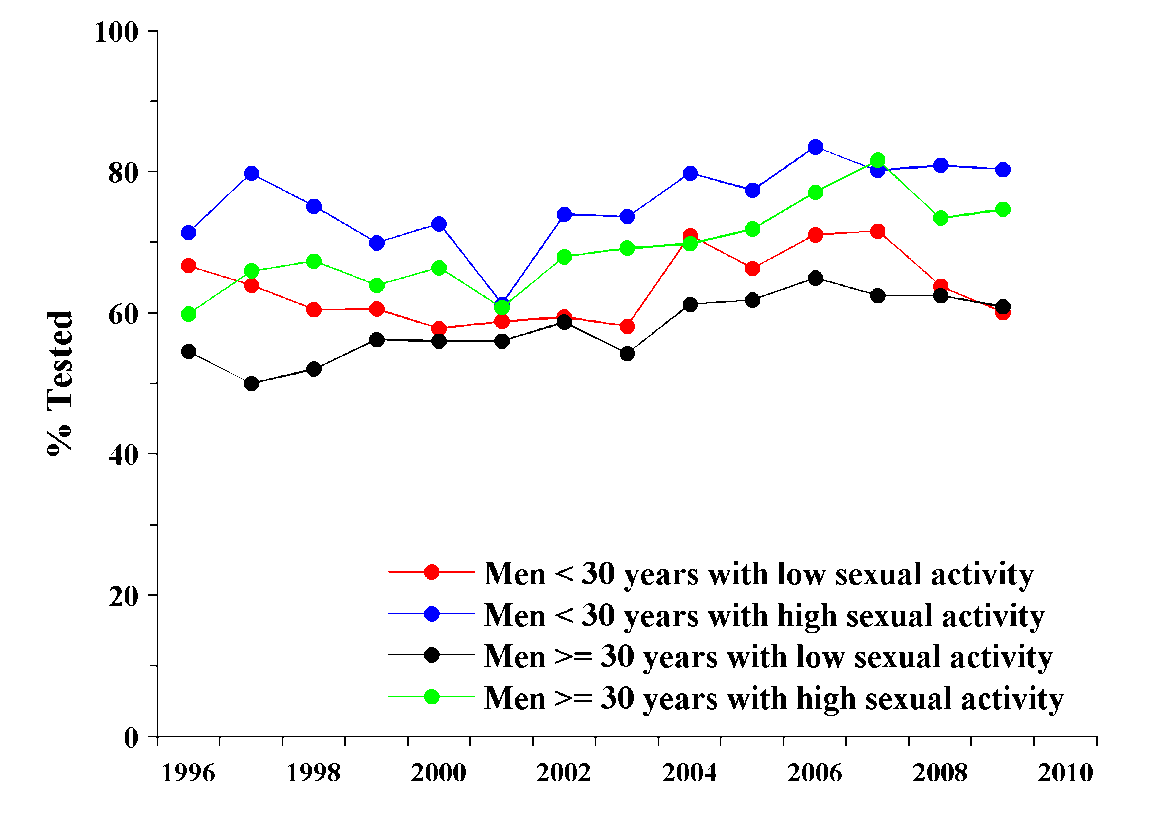  These data suggest that for men who have been tested for HIV, HSA men tend to be tested more frequently than LSA men and that younger men tend to get tested more frequently than older men (though a larger proportion of young men have never been tested as shown in footnote p above). There appears to be a slightly rising trend overall though these rises are relatively small (~10%) and unlikely to be statistically significant. The number of tests carried out in the model population each year per 100 people is shown in Figure S1(c).  v: The sensitivity of a HIV test is dependent on how long someone has been infected with HIV when they get tested. There is a window period where an infected person is less likely to be diagnosed with HIV if they are tested too soon after becoming infected. The assumed sensitivity of a standard HIV test is shown below:  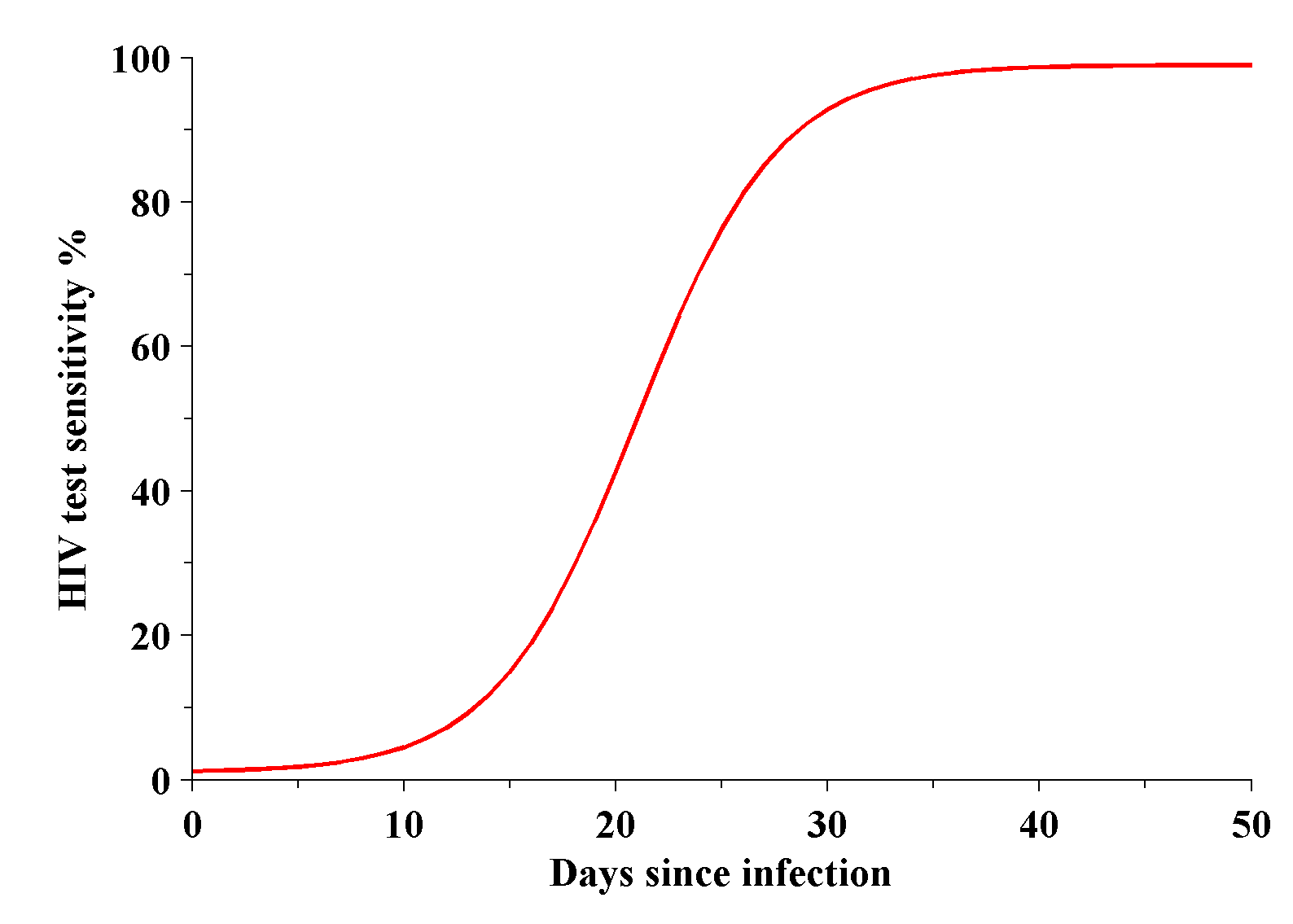  We assume an initial test sensitivity of 1% and a maximum sensitivity of 99%. The length of the window period for a test is quantified by doubling the number of days post infection for the sensitivity to reach 50%, for an Enzyme immunoassay 4th generation test this is assumed to be 42 days.  w: The proportion of men who begin ART with higher CD4 counts is thought to have changed substantially since 1996. Initially it was thought that HIV should be hit hard and early so a relatively high proportion of HIV infected men with higher CD4 counts began treatment soon after 1996 when HAART became available. This proportion is then expected to drop due to problems with the development of resistance to treatment. In recent years treatment has started to again be initiated earlier due to improvements in ART regimens and policy guidelines. This could be modeled with a function that has a “bowl” shape for the period 1996 to 2009. However, given the lack of data for initiation of treatment and that the proportion of HIV positive gay men who are on ART has not changed substantially, shown below [[1](#_ENREF_1)], we assume a constant rate for starting treatment at higher CD4 counts which is calibrated to the available data on the proportion of HIV+ gay men on treatment.  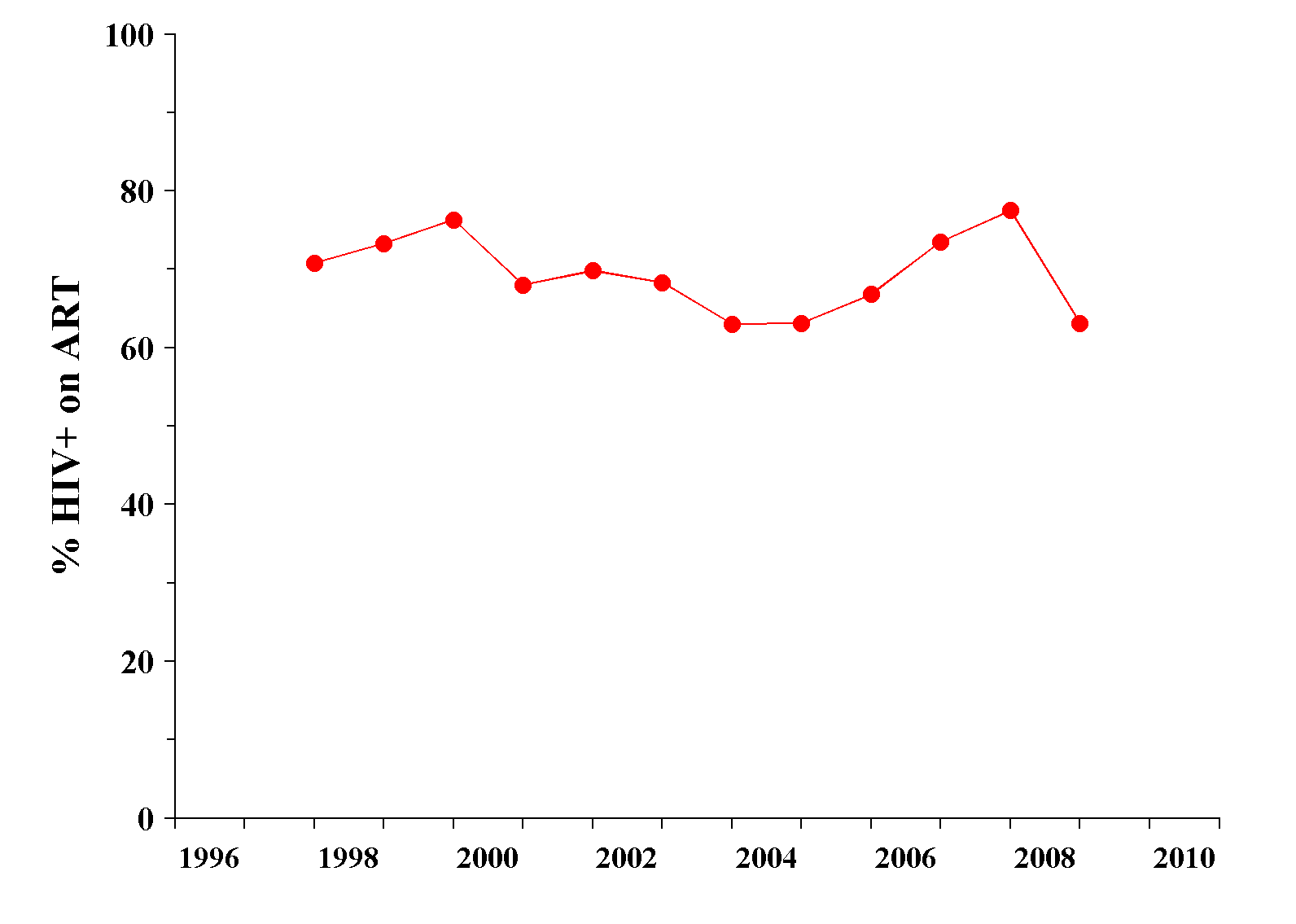  The resulting proportion of the HIV-positive men on ART in the model population is shown in Figure S1(d).  x: In the model this parameter is used to represent the proportion of men on ART who have detectable viral load. From the SGCPS there are data giving the proportion of men on ART with a detectable viral load for the period 2003 to 2009 this is shown as the solid line in the figure below [[1](#_ENREF_1)] (for the period up to 2003 we assume that the proportion is the same as the 2003 value):  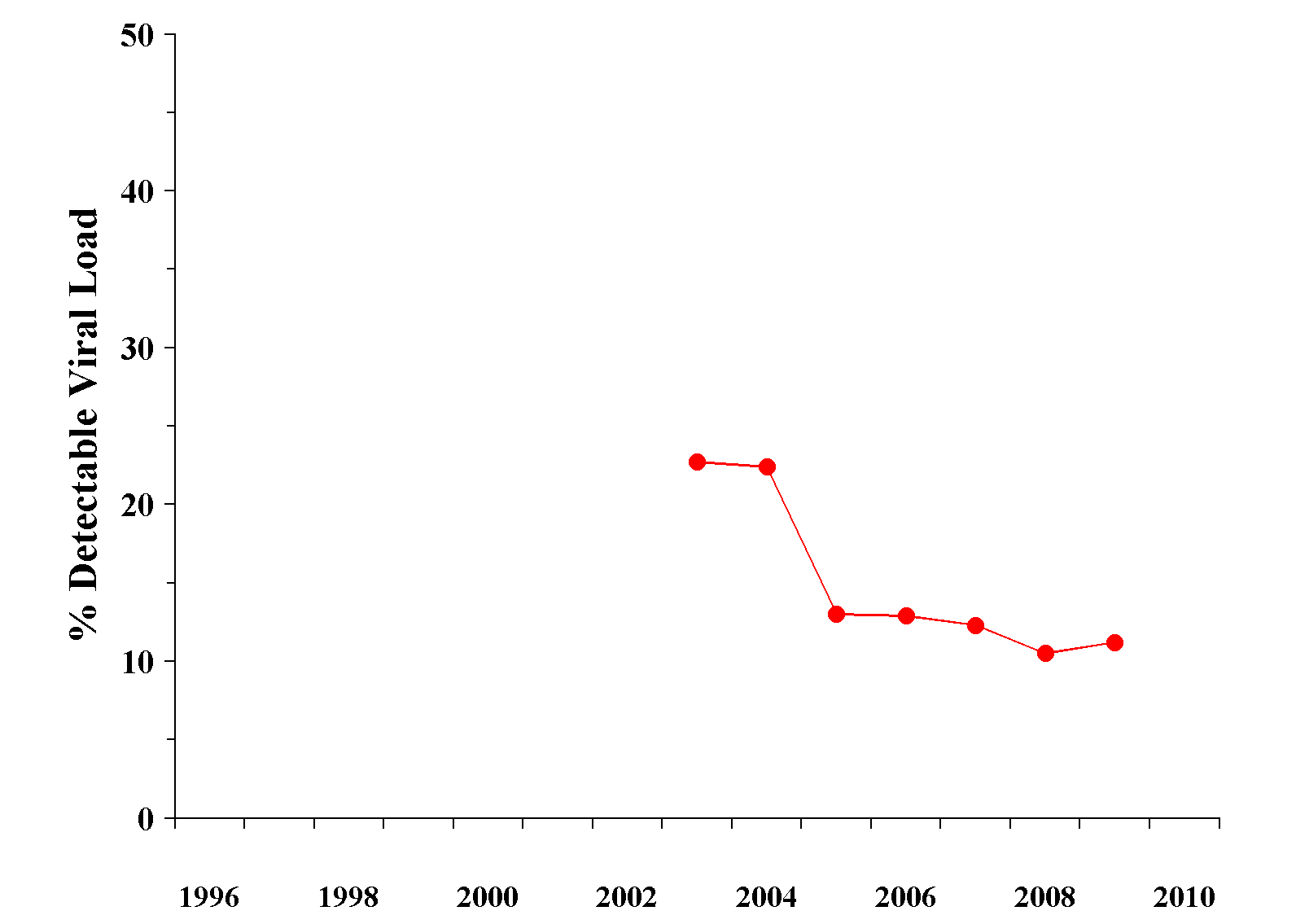  The resulting level of treatment failure at any one time in the model population is shown in Figure S1(e). | | | |
