# Supplementary material for: Increased HIV Testing Will Modestly Reduce HIV Incidence among Gay Men in NSW and Would Be Acceptable if HIV Testing Becomes Convenient
Source: PLoS One. 2013 Feb 15;8(2):e55449. doi: 10.1371/journal.pone.0055449 (PMC3574096; doi:10.1371/journal.pone.0055449)
Supplement: Table S5 — Likelihood of testing (more frequently) among non HIV-positive men (Survey 2). Number of responses recorded with percentage in brackets. (DOCX) [file pone.0055449.s007.docx]

**Table S5:** Likelihood of testing (more frequently) among non HIV-positive men (Survey 2). Number of responses recorded with percentage in brackets

|  | Men tested in previous 12 months N=165 | Men not tested in previous 12 months N=68 | Men reporting no UAIC N=158 | Men reporting UAIC N=75 | TOTAL  N=233 |
| --- | --- | --- | --- | --- | --- |
| Very unlikely | 5 (3.0) | 1 (1.5) | 5 (3.2) | 1 (1.3) | 6 (2.6) |
| Unlikely | 23 (13.9) | 6 (8.8) | 19 (12.0) | 10 (13.3) | 29 (12.4) |
| Likely | 67 (40.6) | 24 (35.3) | 61 (38.6) | 30 (40.0) | 91 (39.1) |
| Very likely | 45 (27.3) | 15 (22.1) | 36 (22.8) | 24 (32.0) | 60 (25.8) |
| NA/NR | 25 (15.2) | 22 (32.4) | 37 (23.4) | 10 (13.4) | 47 (20.2) |
