# Supplementary material for: Increased HIV Testing Will Modestly Reduce HIV Incidence among Gay Men in NSW and Would Be Acceptable if HIV Testing Becomes Convenient
Source: PLoS One. 2013 Feb 15;8(2):e55449. doi: 10.1371/journal.pone.0055449 (PMC3574096; doi:10.1371/journal.pone.0055449)
Supplement: Table S6 — Reasons for delaying or not testing. Number of responses recorded with percentage in brackets. (DOCX) [file pone.0055449.s008.docx]

**Table S6:** Reasons for delaying or not testing. Number of responses recorded with percentage in brackets.

|  | Men tested in previous 12 months N=165 | Men not tested in previous 12 months N=68 | Men reporting no UAIC N=158 | Men reporting UAIC N=75 | TOTAL  N=233 |
| --- | --- | --- | --- | --- | --- |
| Haven’t done anything risky | 59 (35.8) | 33 (48.5) | 72 (45.6) | 20 (26.7) | 92 (39.5) |
| Having to return another time for the results | 72 (43.6) | 18 (26.5) | 57 (36.1) | 33 (44.0) | 90 (38.6) |
| I haven't enough time | 42 (25.5) | 15 (22.1) | 36 (22.8) | 21 (28.0) | 57 (24.5) |
| I haven't had any illness or symptoms which made me worry | 33 (20.0) | 21 (30.9) | 33 (20.9) | 21 (28.0) | 54 (23.2) |
| I haven't changed partners | 35 (21.2) | 16 (23.5) | 42 (26.6) | 9 (12.0) | 51 (21.9) |
| It's too much hassle | 27 (16.4) | 9 (13.2) | 24 (15.2) | 12 (16.0) | 36 (15.5) |
| I don't like needles | 23 (13.9) | 11 (16.2) | 23 (14.6) | 11 (14.7) | 34 (14.6) |
| I'm afraid I might be told I have HIV | 21 (12.7) | 7 (10.3) | 19 (12.0) | 9 (12.0) | 28 (12.0) |
| It's difficult to get an appointment | 22 (13.3) | 6 (8.8) | 17 (10.8) | 11 (14.7) | 28 (12.0) |
| I am afraid to get tested | 16 (9.7) | 6 (8.8) | 12 (7.6) | 10 (13.3) | 22 (9.4) |
| My doctor doesn't bulk bill | 17 (10.3) | 5 (7.4) | 14 (8.9) | 8 (10.7) | 22 (9.4) |
| I feel embarrassed talking about my sex life to the doctor/nurse | 13 (7.9) | 7 (10.3) | 12 (7.6) | 8 (10.7) | 20 (8.6) |
| I'm afraid of letting other people know if my test shows I have HIV | 13 (7.9) | 5 (7.4) | 9 (5.7) | 9 (12.0) | 18 (7.7) |
| I don't want to know | 7 (4.2) | 5 (7.4) | 6 (3.8) | 6 (8.0) | 12 (5.2) |
| I don't have a doctor I can trust | 8 (4.8) | 4 (5.9) | 9 (5.7) | 3 (4.0) | 12 (5.2) |
| I don't want to have to tell my partners if my test shows I have HIV | 8 (4.8) | 3 (4.4) | 4 (2.5) | 7 (9.3) | 11 (4.7) |
| My doctor doesn't suggest it | 8 (4.8) | 1 (1.5) | 6 (3.8) | 3 (4.0) | 9 (3.9) |
| Feeling that I'm being asked to get tested too often | 7 (4.2) | 2 (2.9) | 8 (5.1) | 1 (1.3) | 9 (3.9) |
| I don't know where to get tested | 2 (1.2) | 4 (5.9) | 4 (2.5) | 2 (2.7) | 6 (2.6) |
| Nothing - I never put off getting tested | 41 (24.8) | 2 (2.9) | 25 (15.8) | 18 (24.0) | 43 (18.5) |
