# Supplementary material for: Increased HIV Testing Will Modestly Reduce HIV Incidence among Gay Men in NSW and Would Be Acceptable if HIV Testing Becomes Convenient
Source: PLoS One. 2013 Feb 15;8(2):e55449. doi: 10.1371/journal.pone.0055449 (PMC3574096; doi:10.1371/journal.pone.0055449)
Supplement: Table S7 — What would encourage more men to test (more often)?. Number of responses recorded with percentage in brackets. (DOCX) [file pone.0055449.s009.docx]

**Table S7:** What would encourage more men to test (more often)? Number of responses recorded with percentage in brackets.

| % | Men tested in previous 12 months N=165 | Men not tested in previous 12 months N=68 | Men not reporting UAIC N=158 | Men reporting UAIC N=75 | TOTAL  N=233 |
| --- | --- | --- | --- | --- | --- |
| Receive test results in 20 minutes | 116 (70.3) | 37 (54.4) | 99 (62.7) | 54 (72.0) | 153 (65.6) |
| Saliva-based testing | 98 (59.4) | 39 (57.4) | 85 (53.8) | 52 (69.3) | 137 (58.8) |
| Self-testing at home | 96 (58.2) | 37 (54.4) | 84 (53.2) | 49 (65.3) | 133 (57.1) |
| Finger prick testing | 88 (53.3) | 38 (55.9) | 80 (50.6) | 46 (61.3) | 126 (54.1) |
| Free test site at community organisation | 90 (54.5) | 30 (44.1) | 79 (50.0) | 41 (54.7) | 120 (51.5) |
| Send own saliva or finger prick specimen directly to laboratory | 78 (47.3) | 26 (38.2) | 65 (41.1) | 39 (52.0) | 104 (44.6) |
| Return for test results the next day | 71 (43.0) | 26 (38.2) | 64 (40.5) | 33 (44.0) | 97 (41.7) |
| Receive results by phone or SMS | 66 (40.0) | 18 (26.5) | 49 (31.0) | 35 (46.7) | 84 (36.0) |
| Free test site at gay venue | 65 (39.4) | 27 (39.7) | 60 (38.0) | 32 (42.7) | 92 (39.5) |
| Return for test results in a few days | 52 (31.5) | 18 (26.5) | 46 (29.1) | 24 (32.0) | 70 (30.1) |
| Receive results by email | 69 (41.8) | 20 (29.4) | 52 (32.9) | 37 (49.3) | 89 (8.2) |
