# Supplementary material for: Increased HIV Testing Will Modestly Reduce HIV Incidence among Gay Men in NSW and Would Be Acceptable if HIV Testing Becomes Convenient
Source: PLoS One. 2013 Feb 15;8(2):e55449. doi: 10.1371/journal.pone.0055449 (PMC3574096; doi:10.1371/journal.pone.0055449)
Supplement: Appendix S1 — Technical description of the modeling methodology, parameterization, and calibration. (DOCX) [file pone.0055449.s010.docx]

**Appendix S1 - Detailed Model Description and Methodology**

This Supporting Information describes specific details of the individual-based HIV transmission and sexual behavior model used to model the population level HIV transmission in NSW gay men. The model tracks every individual and sexual partnership in a population of gay men over time with state variables describing the HIV status, HIV progression, CD4 count, ART status and effectiveness, level of sexual activity, sexual behavior preferences, partnership availability, and current sexual partners updated daily. The size of the simulated population is constant, with individuals entering the population as others age out or die, with the sexual behavior and baseline testing-related behavior of each individual fixed during the course of a simulation (unless changed due to a specific intervention). Our model is calibrated using data from sexual behavior surveys of gay men [[1](#_ENREF_1),[2](#_ENREF_2)] to be representative of HIV surveillance data for the state of New South Wales (NSW), Australia over the period 1996 to 2009. The parameters of the model (appropriate for the context of NSW, Australia) are listed in Tables S1-S4.

**Population demographics**

Our model population is made up of 60,000 gay men consistent with the demographics of the NSW gay populations [[2](#_ENREF_2)]. When the population is initialized, individuals are given an age between 15 and 85 years. Individuals leave the population when they reach an age uniformly distributed between 65 and 85 years or if they die due to HIV infection. When a male leaves the population they are immediately replaced, keeping the population size constant, by another male with an age uniformly distributed between 15 and 25 years.

Individuals in the population are categorized to be circumcised based on probabilistically inferred rates estimated from data on the proportion of males circumcised at birth. When a male enters the population they are classified to be circumcised with a probability equal to the proportion of male newborns circumcised in the year corresponding to their birth (Table S1). It is assumed men are only circumcised at birth unless circumcision is rolled-out as a particular biomedical HIV prevention intervention.

**Sexual partnership dynamics**

The model simulates a dynamic sexual partnership network that is updated daily. In the model each person’s sexual activity is determined by the average number of sexual partnerships they have per year. Individuals may engage in three types of sexual partnerships: regular, casual, and group sex. Regular partnerships are long-term partnerships between two gay men with a duration that is geometrically distributed with a mean of 4 years. Casual partnerships have duration of one day and all men can have a casual partnership concurrently with a regular partnership. Group sex partnerships have the same characteristics as casual partnerships except they occur in a group sex setting.

The distributions of the number of casual partners per year were obtained from the ‘Health In Men’ (HIM) study [[3](#_ENREF_3)]. In these studies, the number of casual partnerships per year is categorized into 1-2, 3-5, 6-11, 12-50, and > 50 casual partnerships every six months. We set the maximum number of partnerships in six months to 60 and randomly assigned a category for the number of sexual partnerships based on this distribution. The actual number of casual partnerships for each individual is then randomly determined uniformly from their assigned category. In our model, gay men who have less than 5 casual partnerships every 6 months are designated ‘low activity’ with the others labeled ‘high activity’. For Australian populations approximately 50% of gay men are low activity [[3](#_ENREF_3)].

The model incorporates group sex activity. Almost all gay men engage in group sex at least once in their lifetime, with many men engaging in it infrequently or once-off [[2](#_ENREF_2),[4](#_ENREF_4)]., In our model the sexual activity of each individual is fixed for the duration of a model simulation. The proportion of HIV-negative gay men who regularly engage in group sex is estimated to be 17% [[1](#_ENREF_1)]. In our model, only high-activity gay men are designated to engage in group sex; thus we randomly assign 34% of HIV-negative high activity men in our model population to engage in group sex so that the overall population proportion agrees with these estimates. When a high-activity individual is randomly assigned to be someone who engages in group sex the number of casual partnerships they have per year is decreased by the average number of group sex partnerships for the population (described below). Hence, their overall number of casual sexual partnerships includes the average number of group sex partnerships they have per year. When someone is diagnosed with HIV the number of casual partners they have per six months changes by a value between a 50% reduction and a 10% increase to reflected behavioral changes observed post diagnosis [[5-7](#_ENREF_5)].

In the model simulations, when someone is available to form a casual partnership (see below) another person is selected from the pool of available people. An available individual cycles through potential partners until a suitable partner is found or there are no available partners left (in which case the person does not form a partnership but remains available). Casual partnerships are formed with potential partners with a probability based on their age and HIV serostatus.

A recent analysis of the HIV epidemic in Australian gay men suggested that there is assortative mixing of sexual partnerships based on the age of partners [[8](#_ENREF_8)]. Dividing the population into five-year age bands, this analysis estimated that 30% of a person’s casual partners are from their own five-year age group. To reflect these estimates the probability that a partnership is formed between an individual and a candidate partner is equal to 0.3 if the potential partner is in the same age group and equal to 0.7 if they are in another age group. Another factor affecting the formation of sexual partnerships is the HIV serostatus of each partner. Recent social research suggests that gay men are selecting sexual partners with the same reported HIV serostatus as themselves to reduce the risk of HIV transmission or to engage in anal intercourse without condoms. This practice known as serosorting is difficult to quantify and incorporate into the model but affects the likelihood that two gay men will form a sexual partnership. What is required in the model is the proportion of HIV-positive and HIV-negative men who only engage in anal intercourse with partners with the same serostatus. However, the main source of data for this behavior is UAI with casual partners of the same reported serostatus. The HIM and Positive Health (PH) cohorts [[3](#_ENREF_3)] collected data on the proportion of HIV negative men that had UAI with casual partners who disclosed they were HIV negative and the proportion of HIV positive men who have UAI with casual partners who disclosed they were HIV positive [[3](#_ENREF_3)] (Table S2). This data does not describe the proportion of men who always choose their sexual partners based on their serostatus but by using these reported proportions for the proportion who serosort the model will match the number of UAI acts between partners that are HIV seroconcordant, matching the behavioral data. However, it means our model likely over represents the level of serosorting in the population. Nevertheless, given the assumed high protection provided by condoms (approximately 95% reduction in risk per-act; see Table S4) the majority of infections will occur during UAI within regular and casual partners meaning our model will accurately reflect HIV transmission in the gay community. The proportion of HIV-negative and HIV-positive men who serosort for partners is forced to be a particular value each year for calibration purposes to match annual behavioral data from the HIM and PH cohorts [[3](#_ENREF_3)]. This is done by changing the proportion of men who serosort at the time step corresponding to the beginning of the year. If the required value for HIV-negative/positive men is higher than the current proportion then a proportion of men who are not serosorters become serosorters. Similarly, if the required value is less than the current proportion then a proportion of men who do serosort are reassigned to no longer serosort.

If neither of the two people in a casual partnership also has a regular partner then the partnership can become regular with probability 0.35. This is calibrated so that the overall probability of an individual being in a regular partnership is 38% to match behavioral data [[1](#_ENREF_1)]. When someone is in a regular partnership they are still available to form a casual partnership. Iin the model individuals can only have one casual partnership per day unless they are engaging in a group sex session.

From the population of gay men available to engage in group sex, groups of males are formed.

The size of these groups $g_{s}$ is given by a generalized Pareto distribution with probability distribution function

$$f(x)= \left( \frac{1}{\sigma} \right)e^{-\left( x-\theta\right)/\sigma}$$

for $x>\theta$ where $\sigma=1.9$ and$\theta=3$. These parameters are set so that the average and median group size is 4.4 and 4 respectively, matching available behavioral data [[2](#_ENREF_2),[4](#_ENREF_4)]. The average number of sexual partnerships $p_{g}$ formed by each individual in the group is uniformly selected from between 1 and${\min(g}_{s}-1, 10)$. Within a group, casual partnerships are formed randomly with a probability equal to$\min\left( 1,p_{g}/(g_{s}-1) \right)$. Given the distribution for the group size, the average number of group sex partners a gay man who engages in group sex has per year is approximately 10. We assume that serosorting does not occur in group sex partnerships. After someone engages in group sex there is a gap time where they are not available to engage in group sex. This gap time for each individual is uniformly distributed between 0 and ${730}/{n_{g}}$ days where $n_{g}$ is the average number of group sex sessions an individual has per year. **Sexual behavior within partnerships**

We assume HIV is only transmitted during anal intercourse within a discordant partnership. The probability of anal intercourse during a day in a regular partnership is given by${f_{a}^{r}}/7$ where $f_{a}^{r}$ is the average number of anal acts per week. For casual and group sex partnerships there is a probability of once off anal sex during the partnership.

In the model, the probability of a condom being used during anal sex is dependent on the serostatus of each partner and whether they have disclosed their serostatus (Table S2). Condom usage is more likely if both partners disclose and are serodiscordant. Males in the model population are categorized to disclose to their partners depending on the type of relationship. Note that men disclose their known HIV status, which corresponds to their HIV infection status at the time of their last HIV test. This means that men who are HIV positive may disclose they are HIV negative. Men are probabilistically assigned to disclose to their regular partners. When a regular partnership is formed the partnership is classified to be disclosed if both partners are assigned to disclose, otherwise it is undisclosed. This disclosure status remains fixed for the duration of the partnership. For casual/group partnerships men are categorized to always disclose, sometimes disclose, or never disclose their known HIV status to their casual/group partners. We assume the same disclosure rates for casual and group partnerships with men categorized to sometimes disclose assumed to disclose their known HIV status to their casual/group partners 20% of the time. Similar to serosorting, the proportion of men in each of these disclosure categories is forced in the model to match annual behavioral data from the Sydney Gay Community Periodic Surveys (SGCPS) [[1](#_ENREF_1)]. This is done by changing the proportion of men who always and never disclose at the time step corresponding to the beginning of the year. For men who always disclose the current proportion in the population is compared to the proportion reported in the SGCPS [[1](#_ENREF_1)]. If the datum value is higher than the current proportion then a proportion of men who sometimes disclose are changed to always disclose to match the datum value. If the datum value is less than the current proportion then a proportion of men who always disclose are changed to sometimes disclose. A similar process occurs for those who never disclose with an appropriate proportion of sometimes disclose men changing to never disclose and vice versa to match the datum value.

During anal intercourse one partner takes the insertive position and the other takes the receptive position. If a condom is used then each partner takes their preferred sexual position. In the case where both partners have the same preferred position the HIV-negative partner’s preference takes precedence. If both partners have no position preference then the HIV negative partner has an equally likely chance of being the receptive partner. When a condom is not used unprotected anal intercourse occurs and the sexual position the HIV negative partner takes depends on the disclosed serostatus of their partner with their position determined probabilistically (thus the model incorporates strategic positioning). If the HIV negative partner takes the receptive position during UAI then the insertive partner may withdraw prior to ejaculation to lower the risk of HIV transmission.

**HIV transmission and clinical characteristics**

The transmission of HIV within a discordant partnership depends on the frequency of anal intercourse within a partnership and the probability of HIV transmission during anal intercourse which is based on the sexual position of the HIV negative partner, the stage of infection for the HIV positive partner, and whether a condom has been used effectively during each act. We do not consider the transmission of STIs in the model focusing solely on HIV transmission. The effect of a background level of STIs in the population is encapsulated in the baseline HIV transmission probabilities (for insertive and receptive intercourse and for circumcised and uncircumcised men listed in Table S3). Temporal variations in the in STI prevalence are not captured in the model.

For transmission purposes HIV-positive men are categorized to be: in the primary stage of infection; in the chronic stage of infection; have late-stage disease/AIDS; be on effective antiretroviral treatment (ART); or be on treatment but experiencing treatment failure.

The effectiveness of a condom in preventing the transmission of HIV from an infected person to a susceptible partner is denoted by ε. If a condom is used during anal intercourse then the infectiousness β of an infected partner is reduced to$\left( 1-\varepsilon\right) \beta$. In the model, there are four baseline values for β corresponding to the HIV transmission probability from infected men in the chronic infection stage to an HIV-negative man. These values from lowest to highest probability correspond to the HIV negative partner being: i) circumcised and in the insertive position; ii) uncircumcised and in the insertive position; iii) in the receptive position with their partner withdrawing prior to ejaculation; and iv) in the receptive position with their partner ejaculating. HIV transmission is dependent on the viral load of the infected partner [[9](#_ENREF_9)] which is much higher for men in the primary infection stage or with AIDS and much lower in men on effective ART. The model uses a multiplicative factor to change the value of β depending on the infection category of the HIV-positive partner. Men on effective ART are assumed to have a 92% reduction in their infectiousness. This is based on analysis of HIV transmission in discordant heterosexual couples engaging in vaginal intercourse [[9](#_ENREF_9),[10](#_ENREF_10)]. There is a possibility there will be a smaller reduction in transmission for anal intercourse [[11](#_ENREF_11)].As there is no direct data available for transmission from men on ART during anal intercourse we use the values obtained from heterosexual transmission.

**HIV infection progression and antiretroviral treatment**

The progression of HIV infection within an individual is recorded through their CD4 count. We binned the CD4 count for each individual in the population into one of five categories: normal/uninfected CD4; >500; 350 to 500; 200 to 350; and < 200 cells/uL. At infection, individuals are moved into the > 500 cells/$\mu$L category and are given a fixed duration for their time in each of the CD4 count categories. These time periods are randomly assigned uniformly at the time of infection from the ranges specified in Table S3.

Initially HIV-positive individuals are classified to be in the primary stage of infection for transmission purposes. This stage has a fixed duration for each individual randomly assigned between 70 and 110 days. Once their primary stage is finished they are classified to be in the chronic/asymptomatic stage. Individuals with a CD4 count < 200 cells/$\mu$L are classified to have AIDS and the time period for the a CD4 count < 200 cells/$\mu$L stage represents the time until death due to AIDS, which can be prevented by the initiation of ART.

HIV-Infected individuals can go onto ART in any CD4 count stage but the per-day probability of initiating treatment is fixed for each CD4 count category and increases as CD4 count decreases (Table S3). These probabilities are calibrated to match the percentage of HIV infected men on ART in the SGCPS [[1](#_ENREF_1)]. Once an individual begins treatment it is assumed they remain on treatment for the duration of their time in the model population. Upon initiation of effective treatment the CD4 count of the treated individual stops decreasing and will begin to increase over time. Individuals on ART are given a fixed duration for the time taken in each CD4 count category as their CD4 count increases. These time periods are randomly assigned uniformly at the time of ART initiation from the ranges specified in Table S3.

At ART initiation there is a probability of treatment failure occurring. There is also a probability per day of treatment failure any time after effective ART has been established (see Table S3). The reasons for treatment failure, such as poor adherence or a weak combination of drugs, are not recorded in the model. The probability of treatment failure is calibrated in the model to match the percentage of HIV infected men on ART with treatment failure in the SGCPS [[1](#_ENREF_1)]. Men on ART undergoing treatment failure remain at their current CD4 count until they restart effective ART where upon their CD4 count will begin to increase again.

To detect treatment failure, men on ART have their viral load tested every 3 months to one year to reflect clinical practice in Australia. Men found to be experiencing treatment failure have a probability (equal to one minus the probability of treatment failure at initiation) of restarting a new treatment regime or schedule that puts them back on effective ART. The exact treatment regime or schedule used is not recorded in the model.

**HIV testing**

To model HIV testing, individuals are tested randomly each day with a probability per day that depends on the sexual behavior, age, and HIV status of each individual and the intervention simulated. This is done in such a way to quantitatively match the current testing practices described in the SGCPS [[1](#_ENREF_1)]. For HIV testing four sub-populations of gay men are considered. These are: gay men younger than 30 who are low activity; men younger than 30 who are high activity; men older than 30 who are low activity; and men older than 30 who have high sexual activity. Surveys of Australian gay men show that for each of these population categories there is a proportion of the population who have never tested for HIV [[2](#_ENREF_2),[12](#_ENREF_12)]. In the model a proportion of men are assigned to be non-testers for HIV. These proportions are matched to testing data from the SGCPS from 1996 to 2009 [[1](#_ENREF_1)]. Compared to men older than 30 years, there are a higher proportion of men younger than 30 years who have never been tested for HIV. To represent this change in the model we change the testing status of men who never test on the day they turn 30 years old. This is done with a probability set so that the proportion of men older than 30 years who undergo testing matches the data from behavioral surveys [[1](#_ENREF_1)].

For the men in each sub-population who are willing to test for HIV there is a different value for the daily testing probability. This is determined by four parameters: the duration of the testing/screening period$d_{t}$; the proportion of the population tested $p{}_{c}$ during this period (coverage); the frequency of testing (average number of tests) for each individual$f_{t}$ during this period; and the gap time between testing periods$g_{t}$. Each sub-population has different values of$p_{t}$ representing different background testing rates. The percentage of gay men in each sub-population who test for HIV at least once each year is listed in Table S4 and based on behavioral surveys [[1](#_ENREF_1)]. For background testing we set $d_{t}=365$ and $g_{t}=0$ (i.e. gay men can be tested all year every year with no period of no testing). These values of $p_{c}, f_{t}, d_{t},$ and $g_{t}$for each sub-population remain fixed in our model unless a specific screening intervention is targeted at the men in that subpopulation. The probability of being tested during an intervention period is given by $p_{t}={p_{c}f_{t}}/{d_{t}}$for the intervention parameters and reset to the background testing rates during the gap time between intervention periods.

In our model, there is a variation in the sensitivity of a HIV test depending on the time since infection based on clinical data for standard HIV tests. If a HIV-positive man is tested a few days after they became infected the HIV test sensitivity is low and they are unlikely to be diagnosed with HIV. If they are tested a few months after they become infected then the HIV test has a high sensitivity and they are highly likely to be diagnosed with HIV. To model this variation in HIV test sensitivity we use a general logistic equation with an initial test sensitivity of 1% and a maximum sensitivity of 99%. The length of the window period for a test (quantified by doubling the number of days post infection for the sensitivity to reach 50%) is assumed to be 42 days.

**Model initialization, running of simulations, and calibration**

The model was implemented using Matlab^®^ 2010b with each simulation tracking the dynamic sexual network, HIV transmission, and disease progression of HIV-infected individuals. It was calibrated to match the estimated HIV diagnoses among gay men in NSW, Australia from 1996 to 2010.

The model population was initialized by randomly assigning the sexual activity, average number of partners, and treatment seeking status for each individual. Initially there are no partnerships in the population but everyone is available to form partnerships and is susceptible to HIV. The model was initially run for five years, to stabilize the partnership dynamics, prior to randomly assigning 10% of individuals to be infected with HIV. Following this initial five-year period the model was then run for 45 years, with the proportion of men who serosort set to zero and the rest of the model parameters fixed to their 1996 values, to establish a steady state for HIV prevalence and annual diagnoses approximately matching the epidemic in 1996. The resulting model population then represents the initial conditions for simulations from 1996. Using this model population the model was run for the next 15 years (corresponding to the years 1996 to 2010) with time varying parameters and serosorting occurring in the population to track HIV transmission and disease progression.

The median trajectory of 50 model simulations accurately reflected the number and trends in HIV diagnoses in NSW gay men. The 10 simulations that best fit the HIV epidemic data (using a Pearson chi-squared test) were selected to forecast the impact of interventions over the next 10 years (as shown in Figure S1andFigure S2). For each of the best fitting interventions the random number seeds generated in Matlab^®^ were stored so that the first 65 years prior to the introduction of an intervention iwere repeated and direct comparisons between interventions could be made.

**Figure S1.** The proportion of men in the model population who: (A) are circumcised; (B) have never been tested for HIV; (C) have a HIV test each year (estimated by dividing the total number of tests by the size of the population); (D) are HIV-positive and taking ART; and (E) are on ART and have a detectable viral load.


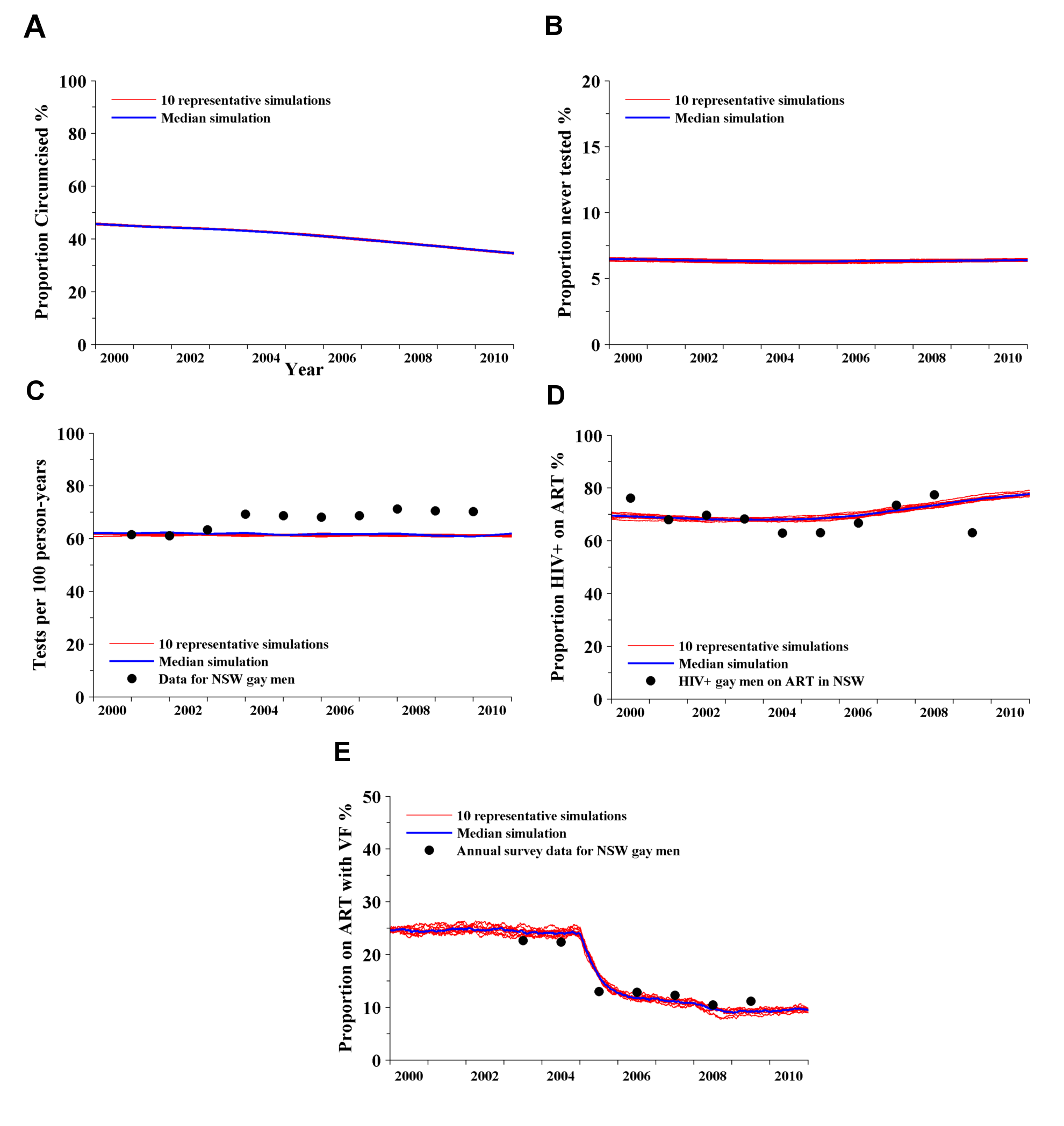


Figure S2. Annual HIV diagnoses from HIV in NSW model and the estimated number of HIV diagnoses in NSW gay men


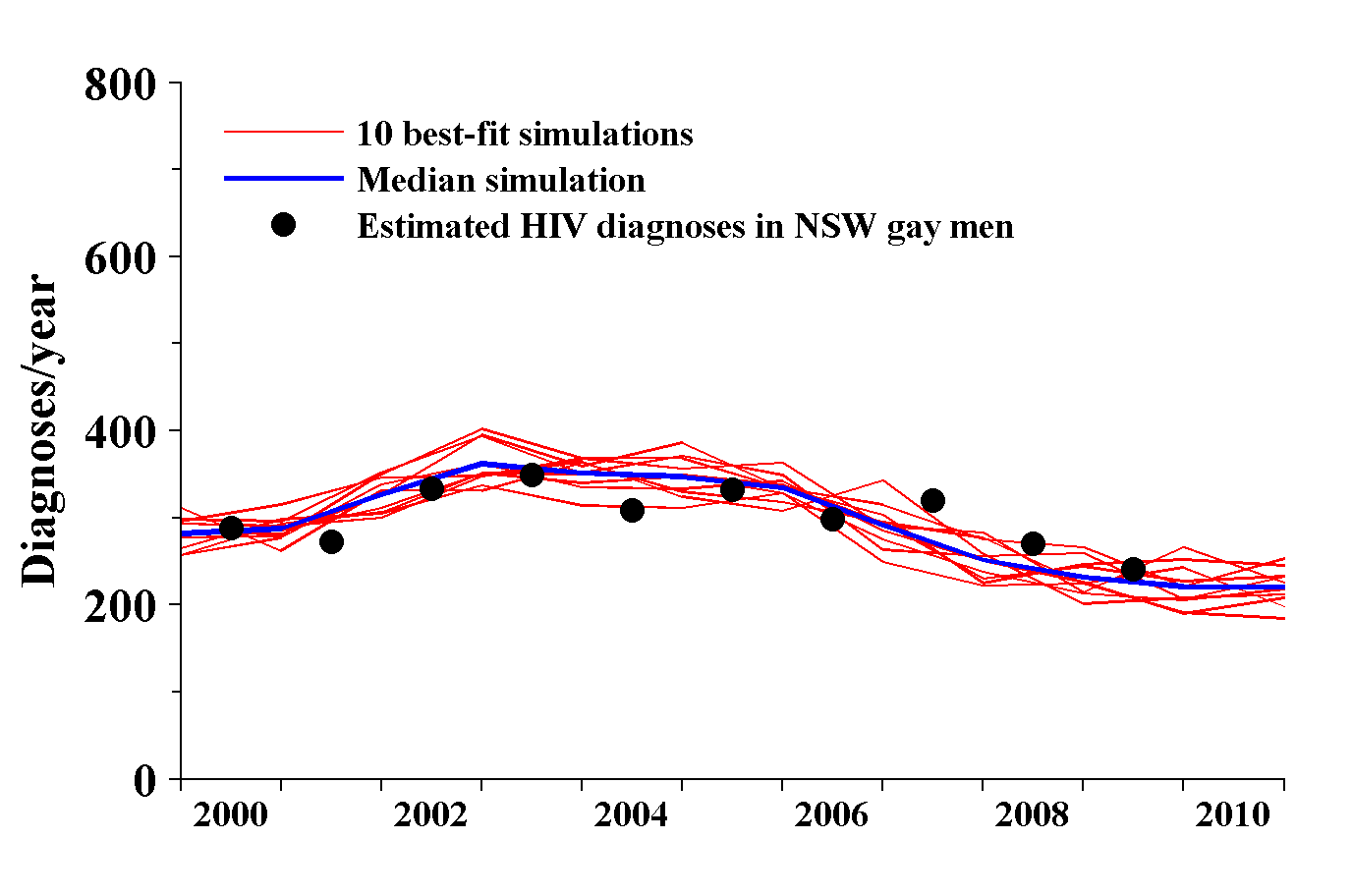


### HIV Testing Interventions

To model the impact of interventions based on HIV testing the values of $p_{c}, f_{t}, d_{t},$ and $g_{t}$ for each age and sexual activity sub-population are changed. Such interventions can be prioritized to specific sub-populations by specifying an age range and a range for the number of sexual partners and changing the coverage and frequency of testing. The particular interventions and their implementation investigated with our model are listed and described below. In addition the model incorporates a probability $p_{n}$that an individual who never tests actually gets tested so that interventions based on rapid or at home testing can be investigated.

1. Increasing testing coverage - To model an increase in the coverage of gay men being tested each year the value of $p_{c}$ is increased from the background value for men willing to test in the target population with $f_{t}, d_{t},$ and $g_{t}$ equal to their background values and the same for each population group.
2. Reducing the number of men who have never tested - An increase in testing coverage can also occur if there is a decrease in the proportion of men who never test. This is modelled by setting$p_{n}>0$ and setting the probability of being tested to be the same as if they were willing testers. Note, if $p_{c}=1$ and $p_{n}=1$ then on average the entire model population will be tested once per year.
3. Increasing test frequency - To model an increase in testing frequency each year the value of $f_{t}$ is increased for men in the target population while $p_{c}, d_{t},$ and $g_{t}$ remain at their background values.
4. Synchronized or ‘blitz’ testing - Modelling of synchronized testing is implemented by setting $d_{t}$ to the duration of the synchronized testing or ‘blitz’ and $g_{t}$ to the time period between testing blitzes. The value of $p_{c}$ is changed to the proportion of men tested during a blitz while $f_{t}$ equals the average number times men are tested in a blitz. For example an intervention that tests 80% of gay men during a one month period twice every year would be implemented by setting $p_{c}=0.8, f_{t}=1, d_{t}=31,$ and $g_{t}=151$. During the period between synchronized testing periods we assume background screening continues to occur.

#

# Baseline parameters for mathematical model

**Table S1** - Model parameters that describe the demographic characteristics of the MSM population in NSW for the period from 1996 to 2010. Parameters are fixed for this period unless available data indicate there have been significant trends as described in the footnotes. The 2010 parameter values are used to represent current conditions.

| **Parameter Description** | | **Values** | **Reference** |
| --- | --- | --- | --- |
| **Demographic** | | | |
| Number of gay men in NSW | | 60,000 | [[2](#_ENREF_2)], a |
| Age of men entering the population | | 15-25 years | b |
| Age of men leaving the population due to old age | | 65-85 years |  |
| Proportion of gay men who are circumcised by age group in 1996 | < 25 years | 59.3% | [[13](#_ENREF_13)], c |
|  | 25-34 years | 69.5% |  |
|  | 35-44 years | 82.6% |  |
|  | > 45 years | 82.6% |  |
| Proportion of gay men entering the population after 1996 who are circumcised | | | d |
| * Model assumption based on discussions with expert stakeholders.  a: The model population is fixed at 60,000 sexually active gay men with young men entering the population as others age out or die.  b: These entry and exit ages are model assumptions based on the age range surveyed in the Sydney Gay Community Periodic Surveys (SGCPS) [[1](#_ENREF_1)]. In the 2007 survey, the median age was 34 years and the maximum age was 91 years.  c: This distribution is assumed for the gay male population in 1996. It is based on data from the HIM cohort which was recorded in 2001-2004 [[13](#_ENREF_13)]. In this cohort 50.5% of < 25 year olds, 59.3% of 25-34 year olds, 69.5% of 35-44 year olds, and 82.6% of > 45 year olds were circumcised. Since, individuals entering the population in 1996 will be aged between 22 and 32 by 2003 we use the 25-34 year old percentage for < 25 year olds in 1996 and similarly for the other age groups. In the model we assume there is no difference in circumcision distribution between HIV negative and positive individuals in 1996.  d: Males entering the population are aged between 15 and 25 years in the model and men entering the population between 1996 and 2009 could have been born between 1971 and 1995. There is limited data available for the proportion of male children in Australia who have been circumcised, however, over this time frame it is known that the circumcision rate has decreased dramatically, likely due to elective neonatal circumcision being no longer available in public hospitals in most states of Australia following changes in medical advice [[14](#_ENREF_14),[15](#_ENREF_15)]. In [[16](#_ENREF_16)] the number of fee-for-service neonatal circumcisions as a percentage of fee- for-service male births from 1979-80 to 1982-83 is published for each state in Australia and overall. Similar data has been published for the period 1994 to 1999 in Western Australia [[17](#_ENREF_17)]. These data are shown below:  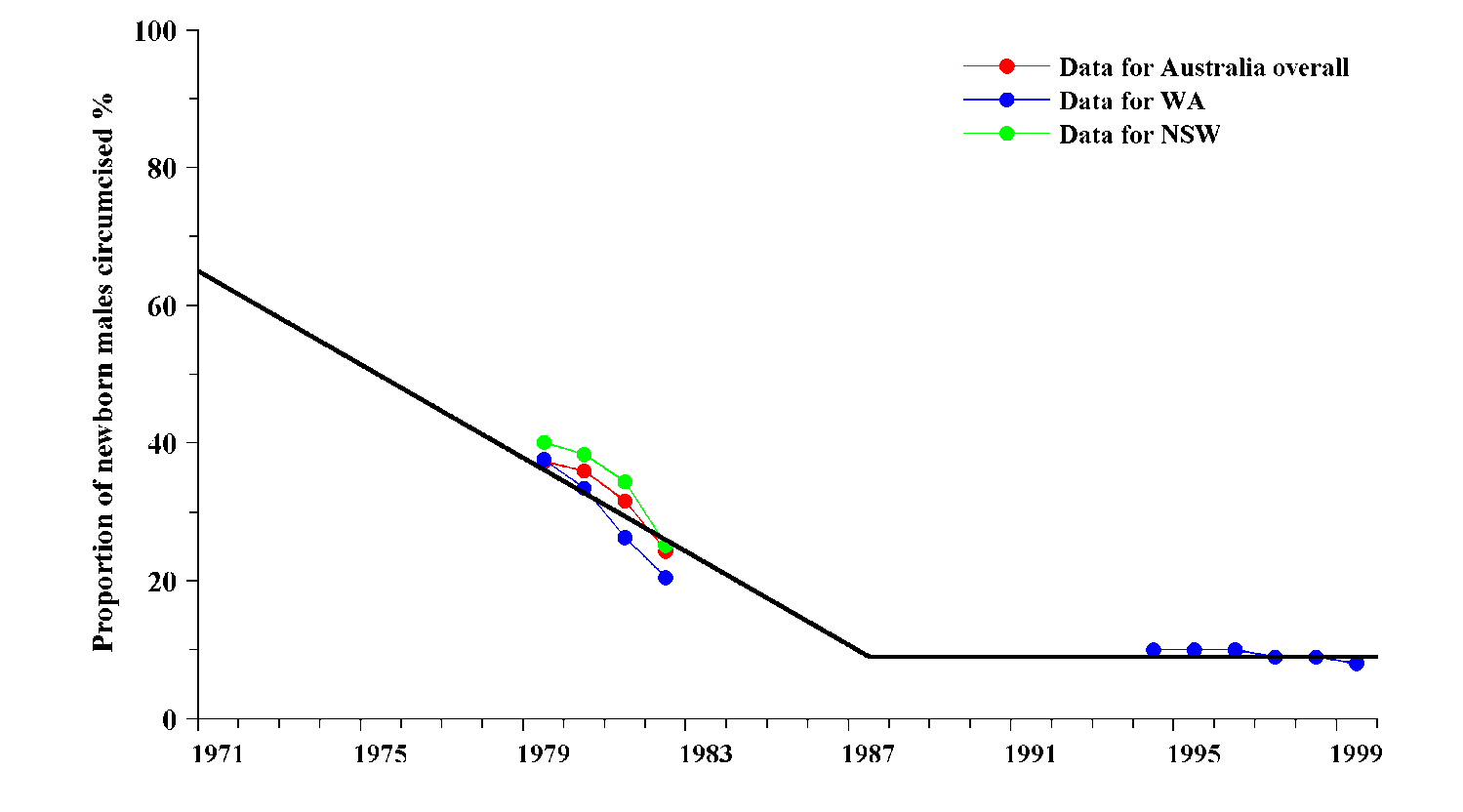  These data show there was a rapid decrease in circumcision rates between 1979 and 1983 which has levelled off to approximately a flat rate slightly less than 10% since 1994. For the model we fitted two straight lines to these data with a linear decrease from 65% in 1971 fitted to the 1979-83 data and a constant rate of 9% fitted to the 1994-99 data in WA. The 65% value for 1971 was chosen based on the percentage of men circumcised in the HIM cohort [[13](#_ENREF_13)] who are aged 25-34 in 2001-2004. The resulting percentage of men in the model population who circumcised is shown in Figure S1(a).  In the model the thick black line in the figure above gives the probability that a new man entering the population is circumcised. | | | |

**Table S2** - Model parameters describing the sexual behavior characteristics of the MSM population in NSW from 1996 to 2010. Parameters are fixed for this period unless available data indicate there have been significant trends as described in the footnotes. The 2010 parameter values are used to represent current conditions.

| **Sexual Behavior** | | | | | | | | | |
| --- | --- | --- | --- | --- | --- | --- | --- | --- | --- |
| Distribution for the number of casual partners gay men have per 6 months (proportion of men in each category) | | 1-3 | | | 26% | | | | [[3](#_ENREF_3)] |
|  |  | 4-10 | | | 21% | | | |  |
|  |  | 11-20 | | | 16% | | | |  |
|  |  | 21-100 | | | 30% | | | |  |
|  |  | 101-120 | | | 7% | | | |  |
| Proportion of partnerships between men in the same 5 year age group | | | | | 30% | | | | [[8](#_ENREF_8)], e |
| Proportion of gay men who engage in group sex | | | | | 17% | | | | [[1](#_ENREF_1)], f |
| Multiplying factor for the change in number of sexual partners post diagnosis of HIV infection (this reflects a possible range from 50% decrease to 10% increase) | | | | | 0.5-1.1 | | | | [[5-7](#_ENREF_5),[18](#_ENREF_18)] |
| Percentage of gay men in a regular sexual partnership | | | | | ~38% | | | | [[1](#_ENREF_1)], g |
| Duration of regular partnerships | | | | | Mean 4 years; 95% range 36 days to 14.7 years | | | | [[2](#_ENREF_2)] |
| Average number of penile-anal acts with a regular partner per week | | | | | 2 (95% range 0-4) | | | | * |
| Average number of penile-anal acts per casual partner/encounter | | | | | 0.7 (range 0-1) | | | | [[1](#_ENREF_1)], h |
| Proportion of partnerships in which HIV serostatus is disclosed | | | | | | | | | i |
| Proportion of men who disclose their HIV infection status and serosort for  regular and casual partners who are seroconcordant | | | | | | | | | j |
| Proportion of sexual acts in which condoms are used for partnerships where the  HIV serostatus of one or both partners is unknown (equal to average condom use) | | | | | | | | | k |
| Proportion of sexual acts in which condoms are used for partnerships that are reported to be | | | | HIV concordant | | 0.2*average | | | l |
|  |  |  |  | HIV discordant | | (1-1.2)*average | | | m |
| Distribution of sexual position preferences | | | | | Insertive only | | | 33% | [[19](#_ENREF_19)] |
|  |  |  |  |  | Receptive only | | | 10% |  |
|  |  |  |  |  | Insertive and receptive | | | 57% |  |
| Sexual position matrix for HIV negative men who engage in UAI based on HIV serostatus of partner |  | | Receptive | | Receptive with withdrawal | | Insertive | | [[19-21](#_ENREF_19)], n |
|  | Partner reports negative | | 28% | | 17% | | 55% | |  |
|  | Partner status unknown | | 17% | | 22% | | 61% | |  |
|  | Partner reports positive | | 6% | | 18% | | 76% | |  |
| *Group sex parameters* | | | | | | | | | |
| Average number of group sex events per year for men who engage in group sex | | | | | 3.5 | | | | [[2](#_ENREF_2)] |
| Median number of men in each group sex event | | | | | Median 4.4 | | | | [[2](#_ENREF_2),[4](#_ENREF_4)] |
| Number of sexual partners in group sex event per person | | | | | Min: 1, Max: 10 | | | | * |
| * Model assumption based on discussions with expert stakeholders.  e: Other modelling work investigating the transmission of HIV in Australian gay men has estimated the level of assortativity of partnerships between gay men in terms of age [[8](#_ENREF_8)]. The results of this work can be interpreted to mean that 30% of a gay man’s partnerships are within their own age group (stratified in age bands of 5 years) with the remaining 70% of their partnerships randomly occurring with men in other age groups.  f: Recent studies of group sex among gay men in Australia suggest that most gay men have engaged in group sex at some time [[2](#_ENREF_2)] with 47% of HIV-negative gay men and 64% of HIV-positive gay men engaging in group sex in the previous 6 months [[1](#_ENREF_1)]. However, a large proportion of gay men only engage in group sex once off or very infrequently. The value here is the estimated value for the proportion of HIV negative gay men who engage in group sex regularly every year. We assume that only high activity gay men engage in group sex, however, for a person who engages in group sex, their total number of casual partnerships also includes their group sex partnerships. In our model the proportions of high activity men who engage in group sex is calibrated so that the overall proportion is equal to the values in the table.  g: In the model a casual partnership can develop into a regular partnership with a probability calibrated so that the proportion of gay men that have a regular partnership at any one time is 38%. This is slightly lower than the approximate 50% reported in the Gay Community Periodic Survey [[1](#_ENREF_1)].  h: Within a casual partnership men may engage in oral sex, anal sex, or both. We only consider anal sex for HIV transmission.  i: In the model gay men are designated to disclose their known HIV serostatus to regular, casual, and group partners. Data from the SGCPS [[1](#_ENREF_1)] gives the proportion of gay men who engage in unprotected anal intercourse (UAI) that always and never disclose their HIV serostatus. For regular partnerships a high proportion of men are assumed to always disclose their serostatus to their regular partners. Given the lack of data for this type of partnership it is assumed that the change in this proportion over time from 1996 to 2009 is constant at 90%.  For casual and group partnerships the proportion of men who engage in unprotected anal intercourse with casual partners (UAIC) and always disclose and who never disclose is reported in the SGCPS [[1](#_ENREF_1)] (this is assumed to be the same for group partnerships). Men are assigned to always disclose, sometimes disclose, or never disclose their serostatus in the model. The change in these proportions over time is shown in the following figure:  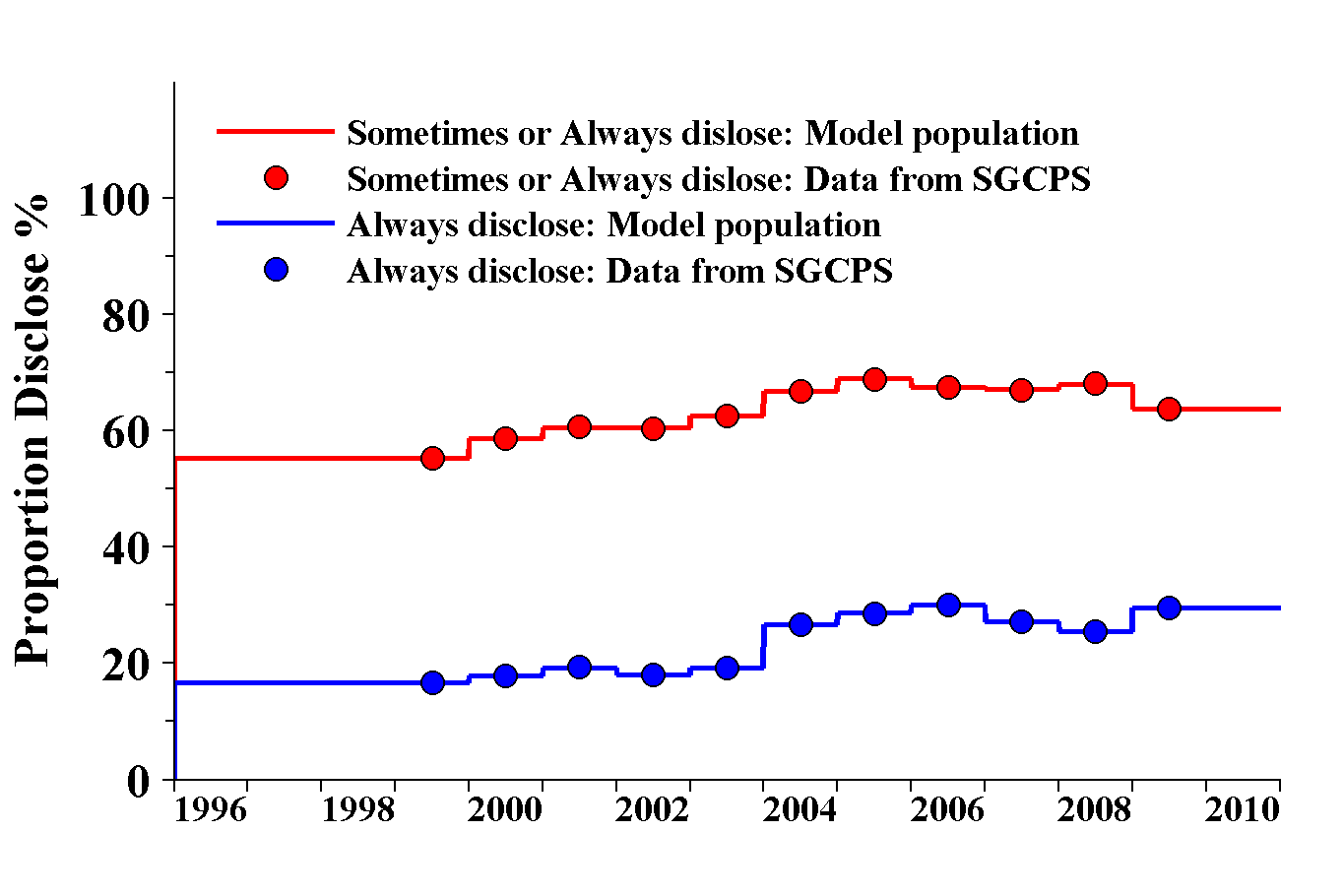  In the model, the proportion of men that always disclose and that never disclose is forced to match the exact data values from behavioral surveys, as shown in the figure above. The proportion of men assigned to sometimes disclose is equal to one minus the sum of the always and never disclose proportions. Men who sometimes disclose are assumed to disclose their HIV status to 20% of their casual/group partnerships. We assume the disclosure rates from 1996 to 1998 are the same as the 1999 levels which is the first year this type of data was recorded.  j: The practice of serosorting is hard to quantify using available data. The HIM and PH cohorts [[3](#_ENREF_3)] record the proportion of men who had UAIC with only seroconcordant casual partners from 2001 to 2006. These data are shown below:  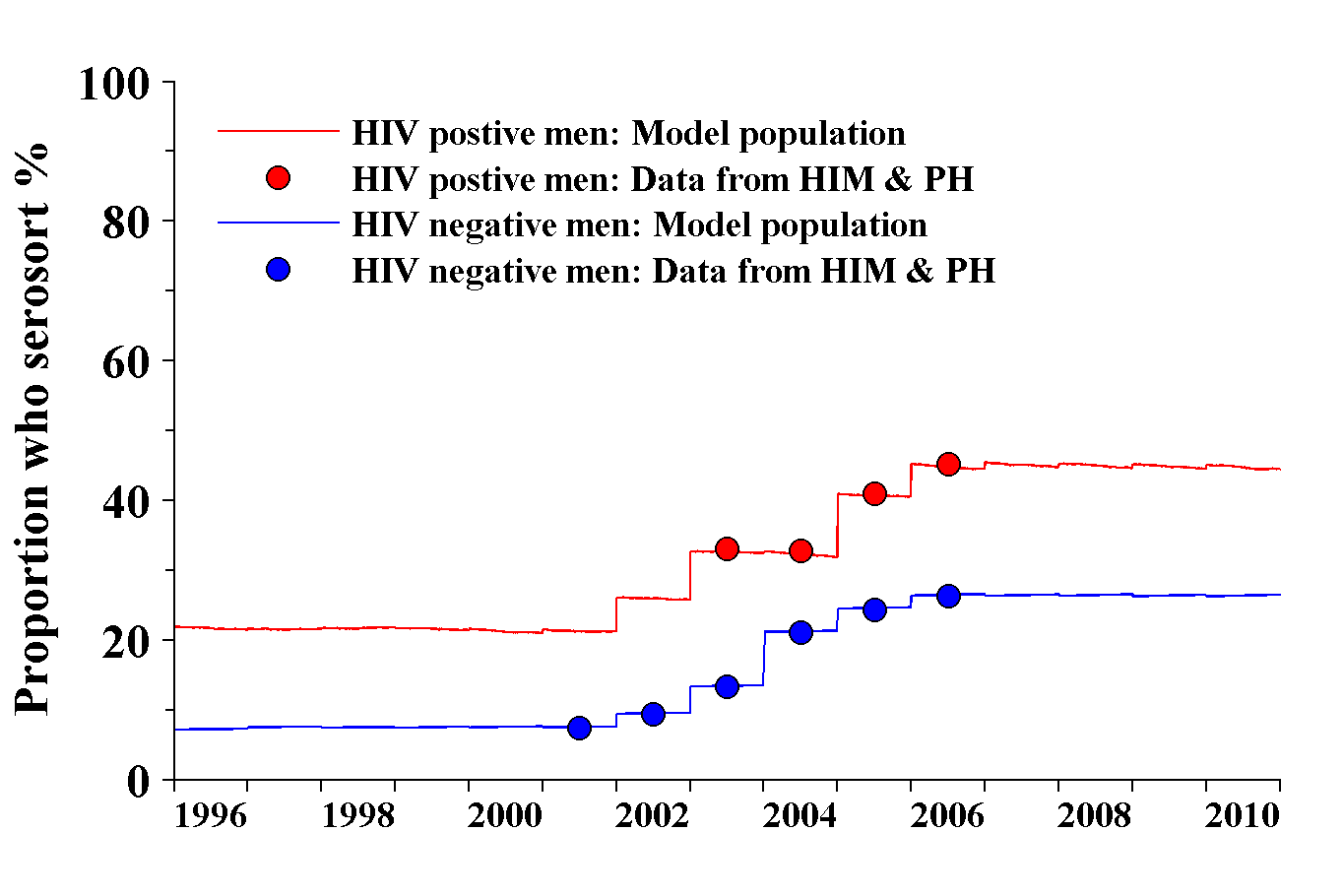  These data suggest that those who engage in UAIC serosort for partners with the same HIV serostatus with HIV positive men more likely to serosort for UAIC partners than HIV negative men and that serosorting for UAIC partners has been increasing over time since 2000. As HIV transmission is most likely to occur during UAI we assume the proportion of men who serosort is equal to the proportions recorded in the HIM and PH cohorts, as explained in the main text of the Supporting Information. Given that HIV transmission is most likely to occur during UAI we assume the proportion of men who serosort is equal to the proportions in the figure above to ensure that the proportion during UAIC matches these data. To determine the values of these proportions for the years when they were not recorded a linear relationship was initially fitted to these data to reflect the likely increase from 1996 to 2009. The level of serosorting prior to 2000 was set at a fixed level equal to the 1996 value. This 1996 value was calibrated so that the number of HIV diagnoses in 1996 matched the number of diagnoses in NSW during that year. After 2006 we assume the proportion of men who serosort is fixed at the 2006 value.    k: In the model condom usage is based on the whether a partnerships is discordant, concordant or unknown in terms of the disclosure of HIV serostatus. The SGCPS record the proportion of gay men who engage in UAI in casual partnerships. This is a reflection of condom use overall in the community and one minus this proportion gives the percentage of men who always use a condom during casual anal intercourse. The proportion of gay men who engaged in UAIC each year is shown below [[1](#_ENREF_1)]:  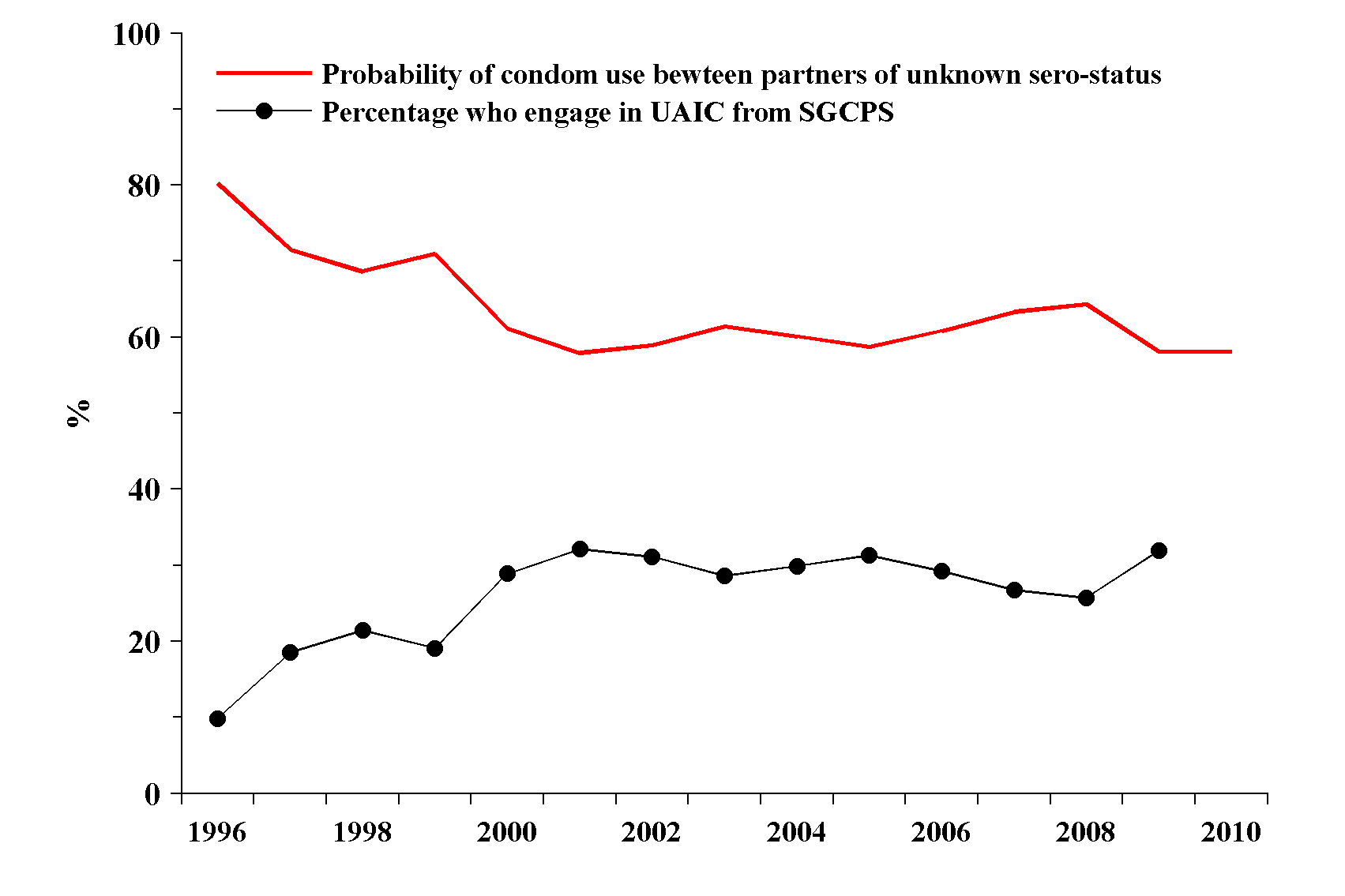  In the model the probability that a condom is used during AI in a partnership where the HIV serostatus of one or both partners is assumed to be 10% less than the proportion of gay men who always use a condom in casual partnerships.  l: The condom usage in partnerships that are thought to be HIV concordant is assumed to be very low. From the SGCPS 65% to 85% of gay men in regular seroconcordant HIV-positive partnerships and seroconcordant HIV-negative partnerships have engaged in UAI, respectively (for 2003 to 2009) [[1](#_ENREF_1)]. This means only 20-30% of men always use condoms in concordant regular partnerships over the multiple episodes of sexual intercourse. For the model we assume that condom usage rates for concordant partnerships is 20% of the rate for partnerships of unknown HIV serostatus. This varies from ~18% to 13%.  m: Condom usage in partnerships that are thought to be HIV discordant is assumed to be very high. From the SGCPS 45% to 39% of gay men in regular serdiscordant partnerships have engaged in UAI (for 2003 to 2009) implying 55 to 65% of men always use a condom in discordant regular partnerships [[1](#_ENREF_1)]. Given that this is over multiple sexual encounters, the probability of using a condom must be high. For the model we assume the probability that a condom is used per act in a discordant partnership is the minimum of 100% and 1.2 times the average probability (described in footnote k).  n: These values are based on a simple analysis of the sexual behaviour of men in the HIM cohort [[3](#_ENREF_3)]. In this cohort there are 1334 HIV negative men and the data presented gives the proportion of UAI acts these men had while in the cohort that were insertive, receptive with withdrawal, and receptive with ejaculation:  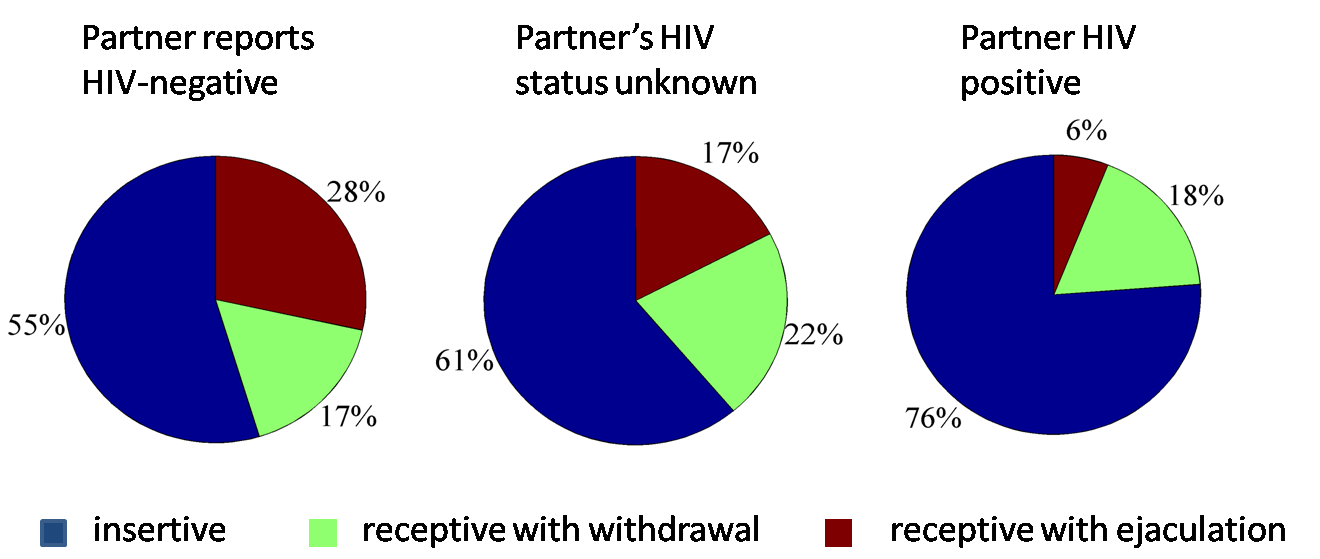 | | | | | | | | | |

**Table S3** - Model parameters describing the HIV biological characteristics of the MSM population in NSW from 1996 to 2010. Parameters are fixed for this period unless available data indicate there have been significant trends as described in the footnotes. The 2010 parameter values are used to represent current conditions.

| **HIV Biological Parameters** | | | |
| --- | --- | --- | --- |
| *Disease Progression for men not on ART* | |  |  |
| Duration of Primary HIV stage | | 70-110 days | * |
| Time taken for HIV infected men to progress from: | CD4 count > 500 cells/μL to CD4 count 350-500 cells/μL | 1.79-4.42 years | [[22](#_ENREF_22)], o |
|  | CD4 count 350-500 cells/μL to CD4 count 200-350 cells/μL | 1.81-2.13 years |  |
|  | CD4 count 200-350 cells/μL to CD4 count < 200 cells/μL | 1.81-2.13 years |  |
| *Disease progression for men on ART* | | | |
| Time taken for HIV infected men on ART with undetectable viral load to progress from: | CD4 count < 200 cells/μL to CD4 count 200-350 cells/μL | 2.33-3.58 years | [[23](#_ENREF_23)], p |
|  | CD4 count 200-350 cells/μL to CD4 count 350-500 cells/μL | 0.90-3.42 years |  |
|  | CD4 count 350-500 cells/μL to CD4 count > 500 cells/μL | 1.07-7.28 years |  |
| *Death rates* | | | |
| Death rate per year for HIV infected men: | with CD4 count > 500 cells/μL and detectable viral load | 0.051% | [[24](#_ENREF_24)] |
|  | with CD4 count between 500 and 350 cells/μL and detectable viral load | 0.128% | [[24](#_ENREF_24)] |
|  | with CD4 count between 350 and 200 cells/μL and detectable viral load | 1% | [[24](#_ENREF_24),[25](#_ENREF_25)] |
|  | with CD4 count < 200 cells/μL and with detectable viral load | 5% | [[24](#_ENREF_24),[25](#_ENREF_25)], q |
|  | with CD4 count < 200 cells/μL and with undetectable viral load | 2% | r |
| *HIV transmission* | | | |
| Baseline per-contact probability of HIV transmission during UAI for insertive acts | Uncircumcised | Circumcised | [[11](#_ENREF_11)] |
|  | 0.42% | 0.074% |  |
| Baseline per-contact probability of HIV transmission during UAI for receptive acts | With withdrawal | With ejaculation | [[11](#_ENREF_11)] |
|  | 0.44% | 0.97% |  |
| Multiplicative change in HIV transmission probability from baseline  if infected partner: | is in primary stage of infection | 5.5 | [[9](#_ENREF_9),[26](#_ENREF_26)] |
|  | has AIDS | 5.5 | [[9](#_ENREF_9),[26](#_ENREF_26)] |
|  | is on effective ART | 0.08 | [[9](#_ENREF_9),[10](#_ENREF_10)] |
|  | is on ART but experiencing treatment failure | 1 | [[9](#_ENREF_9),[10](#_ENREF_10)] |
| * Model assumption based on discussions with expert stakeholders.  o: A summary of the relation between HIV-1 RNA concentration and decline in CD4 count from the prospective study by Mellors et al. [[22](#_ENREF_22)] is given below:   \| Plasma HIV-1 RNA  concentration (copies/mL) \| Mean decrease in CD4+ T cell  count per year (cells/µL) \| \| --- \| --- \| \| ≤ 500 \| 36.3 (30.4, 42.3) \| \| 501-3,000 \| 44.8 (39.1, 50.5) \| \| 3,001-10,000 \| 55.2 (50.7, 59.8) \| \| 10,001-30,000 \| 64.8 (59.6, 70.0) \| \| > 30,000 \| 76.5 (70.5, 82.9) \|   With these data, and assuming that the average viral load is ~104.87 copies per mL for people without treatment, the CD4+ T cell count decreases by an average of 76.5 (70.5, 82.9) every year.   - To progress through the >500 CD4 cell category, we assume that the average CD4 count is 800 cells/μL after the 2-month acute phase of HIV infection and then declines at the constant rate of 76.5 (70.5, 82.9) cells/μL each year. Then the average time to progress through this compartment is 2/12 + 300/(76.5 (70.5, 82.9)) years; that is 4.09 (3.79, 4.42) years. - To progress through the 350-500 and 200-350 CD4 cell categories, we assume an average loss of 150 CD4 cells. Then the average time to progress through this compartment is 150/(76.5 (70.5, 82.9)) years; that is 1.96 (1.81, 2.13) years.   p: Below is a summary of data from [[23](#_ENREF_23)] for changes in CD4 count over time among people who are on effective ART.   \| CD4 count at initiation of ART (cells per μL) \| Time since starting ART (years) \| Curent CD4 (cells per μL) means (95% CI) \| \| --- \| --- \| --- \| \| ≤ 200 \| < 1 \| 76 (53-99) \| \| 1-3 \| 69 (63-76) \| \| 3-5 \| 50 (36-69) \| \| > 5 \| 32 (18-46) \| \| 201-350 \| < 1 \| 129 (91-166) \| \| 1-3 \| 50 (25-74) \| \| 3-5 \| 47 (24-63) \| \| >5 \| 3 (2-44) \| \| > 350 \| < 1 \| 90 (37-144) \| \| 1-3 \| 50 (18-82) \| \| 3-5 \| 17 (-17-51) \| \| > 5 \| 2 (-12-54) \|   We used these data to estimate the average time to progress through our CD4 categories whilst on effective ART. For people with undetectable viral load:  • For CD4 count increases from 0 to 200 cells per µL, average increases of 76 (53-99) cells per µL can be expected during the first year and then 69 (63-76) cells per µL during the second and third years. Therefore, it can be expected to take 2.80 (2.33-3.58) years to progress through this category.  • For CD4 count increases from 200 to 350 cells per µL, we have a 150 CD4 count increase. In this interval, the CD4 count increases by 129 (91-166) cells per µL during the first year and then 50 (25-74) CD4 count during the second year. Therefore, it can be expected to take 1.42 (0.9-3.42) years to progress through this category.  • For CD4 count increases from 350 to 500 cells per µL, then we have a 150 CD4 count increase. In this interval, the CD4 count increases by 90 (37-144) cells per µL during the first year and then 50 (18-82) cells per µL during the second year. Therefore, it can be expected to take 2.20 (1.07-7.28) years to progress through this category.  q: HIV infected men with a CD4 count < 200 cells per µL that are not on treatment are assumed to have AIDS in the model and die within one year unless they start treatment. If they begin treatment but experience treatment failure their death rate is the percentage given in the table.  r: The death rates for men on ART are determined by their CD4 count (which increases over time if they are on effective ART) and is assumed to be the same as for men with detectable viral load. | | | |

**Table S4** - Model parameters describing the clinical characteristics of the MSM population in NSW from 1996 to 2010. Parameters are fixed for this period unless available data indicate there have been significant trends as described in the footnotes. The 2010 parameter values are used to represent current conditions.

| **Clinical Parameters** | | | |
| --- | --- | --- | --- |
| Per-act reduction in HIV transmission by condoms | | 95% | *, s |
| Percentage of the gay population that have never tested for HIV | < 30 years; Low sexual activity | 15% | [[1](#_ENREF_1)], t |
|  | < 30 years; High sexual activity | 8% |  |
|  | >= 30 years; Low sexual activity | 6% |  |
|  | >= 30 years; High sexual activity | 5% |  |
| Percentage of undiagnosed gay men who test for HIV each year in the absence of a specific intervention | < 30 years; Low sexual activity | 55% | [[1](#_ENREF_1)], u |
|  | < 30 years; High sexual activity | 65% |  |
|  | >= 30 years; Low sexual activity | 65% |  |
|  | >= 30 years; High sexual activity | 75% |  |
| Sensitivity of HIV test | | | v |
| *HIV Treatment* | | | |
| Proportion of HIV+ with a CD4 count > 500 cells/μl who go on treatment each year  from 1996 to 2008 | | 0.5% | *, w |
| Proportion of HIV+ with a CD4 count between 500 and 350 cells/μl who go on  treatment each year from 1996 to 2008 | | 20% |  |
| Proportion of HIV+ with a CD4 count between 350 and 200 cells/μl who go on treatment each year from 1996 to 2008 | | 40% |  |
| Proportion of HIV+ with a CD4 count < 200 cells/μl who go on treatment each year from 1996 to 2008 | | 60% |  |
| *Treatment failure* | | | |
| Proportion of men that start ART who experience treatment failure at initiation | | | x |
| Proportion of men on ART who experience viral rebound each year | | 5% | [[27](#_ENREF_27)] |
| *Viral load testing* | | | |
| Time period between viral load tests for HIV positive men on ART to detect if treatment failure has occurred | | 3 months-1 year | * |
| * Model assumption based on discussions with expert stakeholders.  s: This value is assumed based on estimates of condom effectiveness and data on condom breakage and slippage during anal intercourse between MSM. A systematic review of condom effectiveness in partnerships found consistent condom use reduced HIV incidence by 80%—where consistent use is using a condom for all acts of penetrative vaginal intercourse [[28](#_ENREF_28)]. The per-act effectiveness has to be greater than this 80% estimate for consistent condom use. A recent study reported breakage, slippage, and partial use errors occur frequently in MSM partnerships in the US [[29](#_ENREF_29)]—and we would expect similar results for Australian MSM. In the study, out of 1650 condoms used, breakage and slippage occurred in 3.4% of acts and partial use errors (delayed use or early removal) occurred in 11.2% of acts (14.6% overall). The effect of these problems with condoms on transmission is unknown—breakage, slippage and removal before ejaculation could result in no protection but delayed use might only reduce protection slightly. These results reflect previously published results where 1% to 8% of protected acts had condom breakage or slippage during heterosexual intercourse (depending on experience) [[30](#_ENREF_30)]. Based on this data for condom failure and the effectiveness estimate for consistent condom use we assumed a 95% reduction in per-act HIV transmission by condoms.  t: From the SGCPS there is a proportion of the population that has never tested for HIV. From 1996 to 2009 the rates for highly sexually active (HSA) (defined to be those with more than 10 casual partners every 6 months) and low sexual activity men (LSA) (defined to those who have less than 10 sexual partners every 6 months) gay men who are younger and older than 30 years of age are shown below [[1](#_ENREF_1)]:  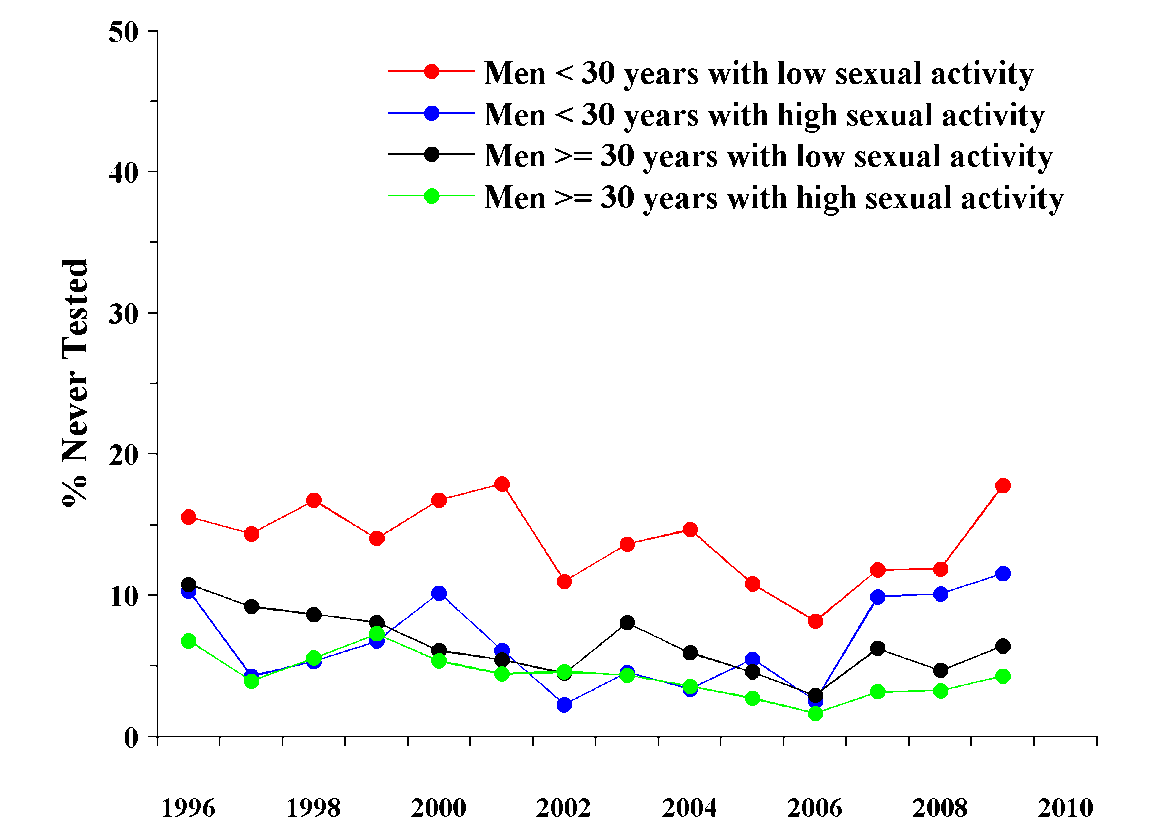  These data show there is a relatively constant level of men who have never tested for HIV with a larger proportion of younger LSA gay men having never tested for HIV (~15%) compared to the rest of the population (5-10%). In the model we assign proportions of men in each population category to never test for HIV with a fixed probability. When younger men who have never tested turn 30 there is a probability that they will become available to be tested for HIV to match the proportions for older men. The overall proportion of men who have never been tested for HIV in the model population is shown in Figure S1(b).  u: The probability that a HIV negative individual is tested for HIV in a given year is based on the proportion of gay men who tested for HIV in the previous 12 months in the SGCPS. For men who have previously tested for HIV the percentage who tested in the previous 12 months from 1996 to 2009 is shown below [[1](#_ENREF_1)]:  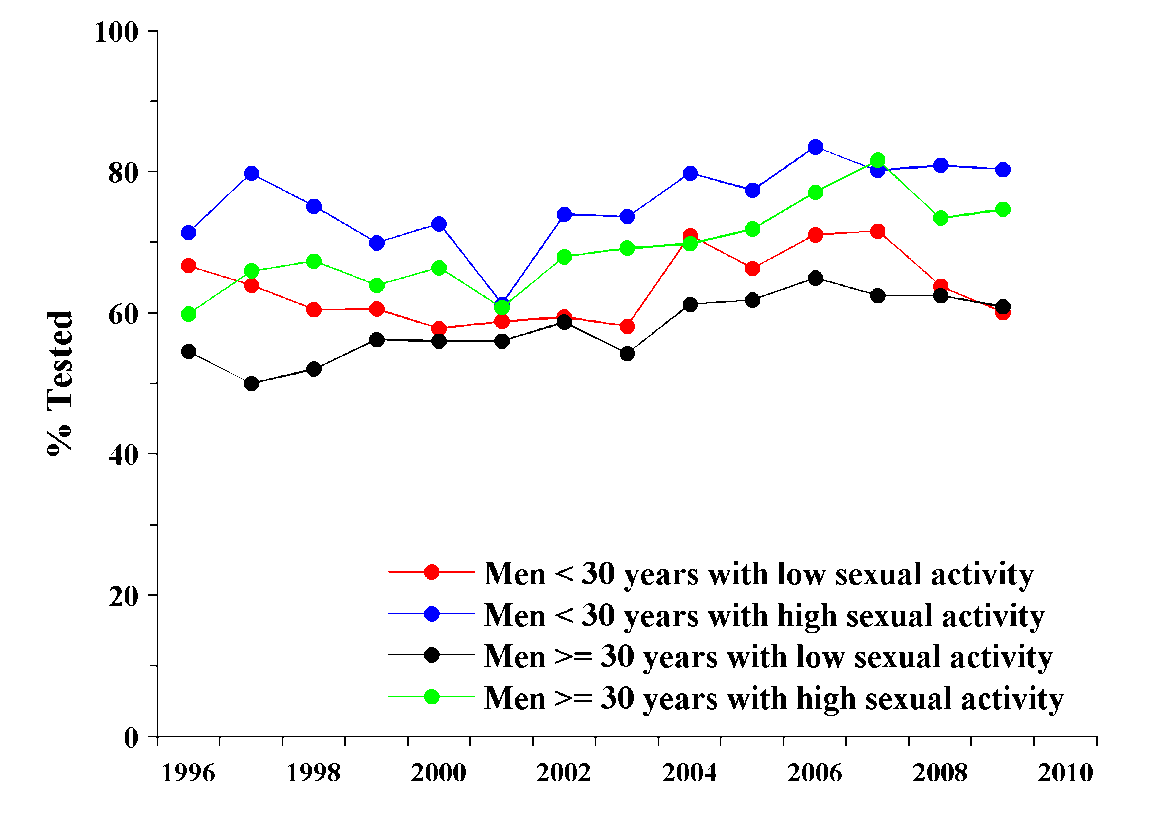  These data suggest that for men who have been tested for HIV, HSA men tend to be tested more frequently than LSA men and that younger men tend to get tested more frequently than older men (though a larger proportion of young men have never been tested as shown in footnote p above). There appears to be a slightly rising trend overall though these rises are relatively small (~10%) and unlikely to be statistically significant. The number of tests carried out in the model population each year per 100 people is shown in Figure S1(c).  v: The sensitivity of a HIV test is dependent on how long someone has been infected with HIV when they get tested. There is a window period where an infected person is less likely to be diagnosed with HIV if they are tested too soon after becoming infected. The assumed sensitivity of a standard HIV test is shown below:  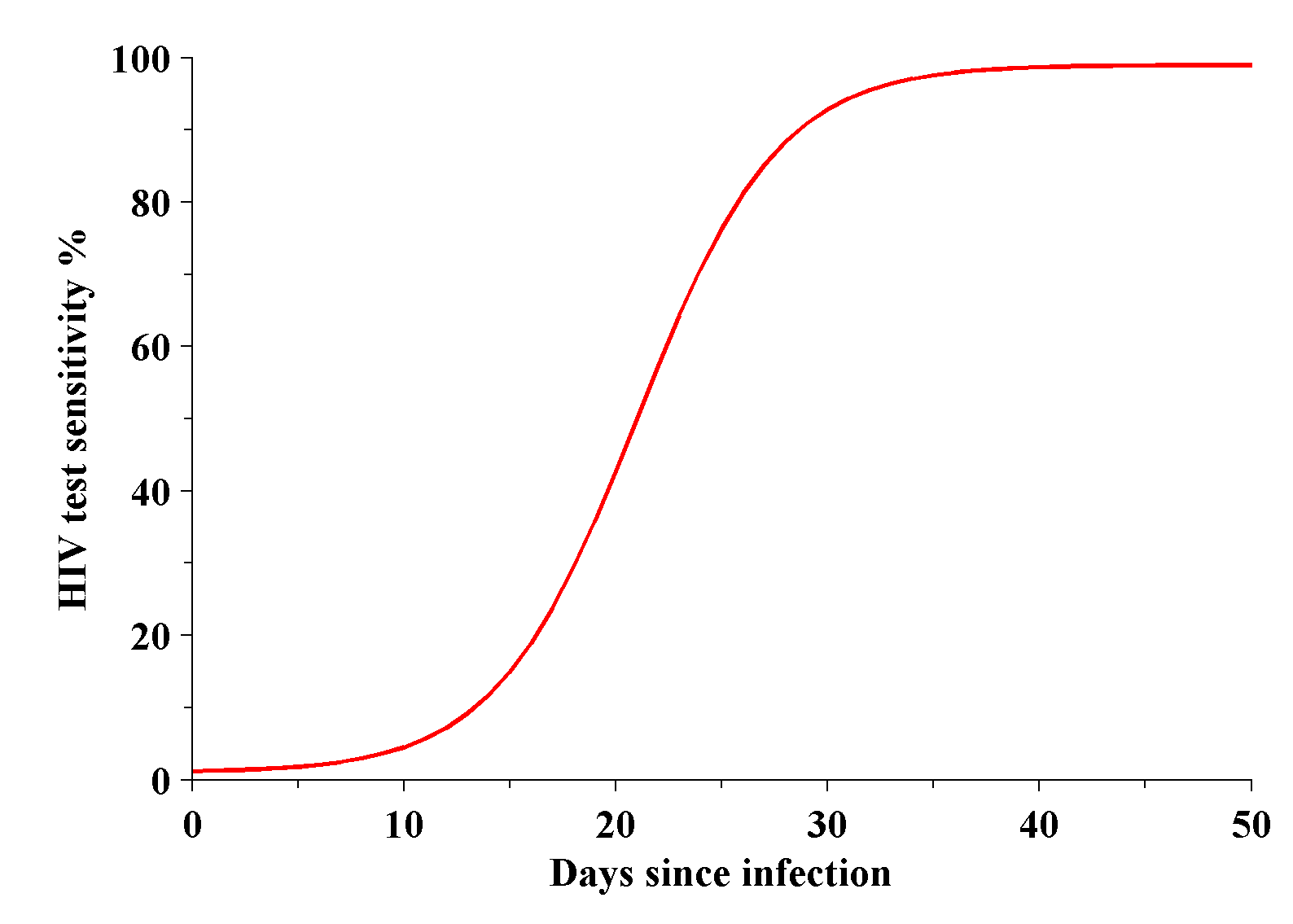  We assume an initial test sensitivity of 1% and a maximum sensitivity of 99%. The length of the window period for a test is quantified by doubling the number of days post infection for the sensitivity to reach 50%, for an Enzyme immunoassay 4th generation test this is assumed to be 42 days.  w: The proportion of men who begin ART with higher CD4 counts is thought to have changed substantially since 1996. Initially it was thought that HIV should be hit hard and early so a relatively high proportion of HIV infected men with higher CD4 counts began treatment soon after 1996 when HAART became available. This proportion is then expected to drop due to problems with the development of resistance to treatment. In recent years treatment has started to again be initiated earlier due to improvements in ART regimens and policy guidelines. This could be modeled with a function that has a “bowl” shape for the period 1996 to 2009. However, given the lack of data for initiation of treatment and that the proportion of HIV positive gay men who are on ART has not changed substantially, shown below [[1](#_ENREF_1)], we assume a constant rate for starting treatment at higher CD4 counts which is calibrated to the available data on the proportion of HIV+ gay men on treatment.  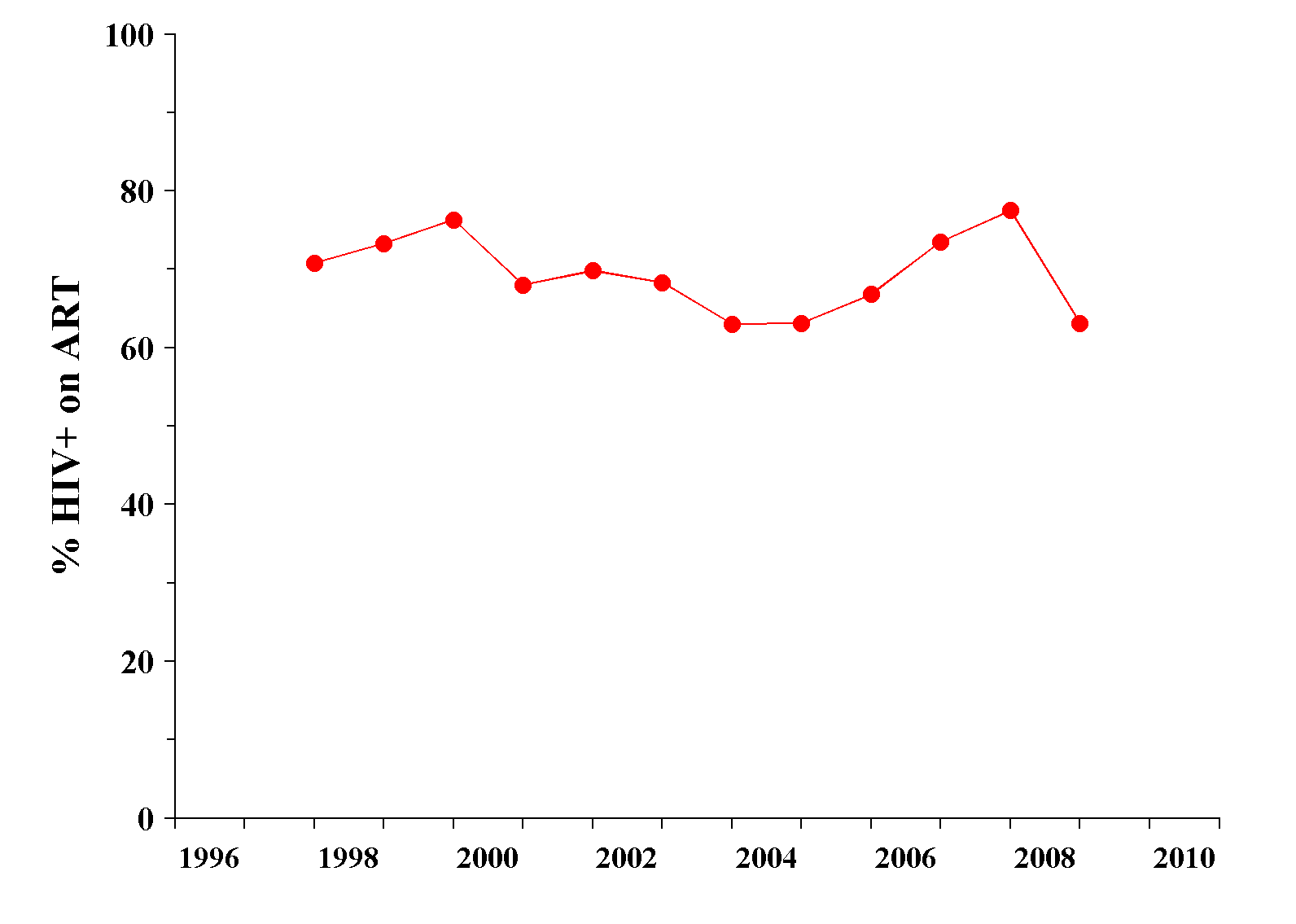  The resulting proportion of the HIV-positive men on ART in the model population is shown in Figure S1(d).  x: In the model this parameter is used to represent the proportion of men on ART who have detectable viral load. From the SGCPS there are data giving the proportion of men on ART with a detectable viral load for the period 2003 to 2009 this is shown as the solid line in the figure below [[1](#_ENREF_1)] (for the period up to 2003 we assume that the proportion is the same as the 2003 value):  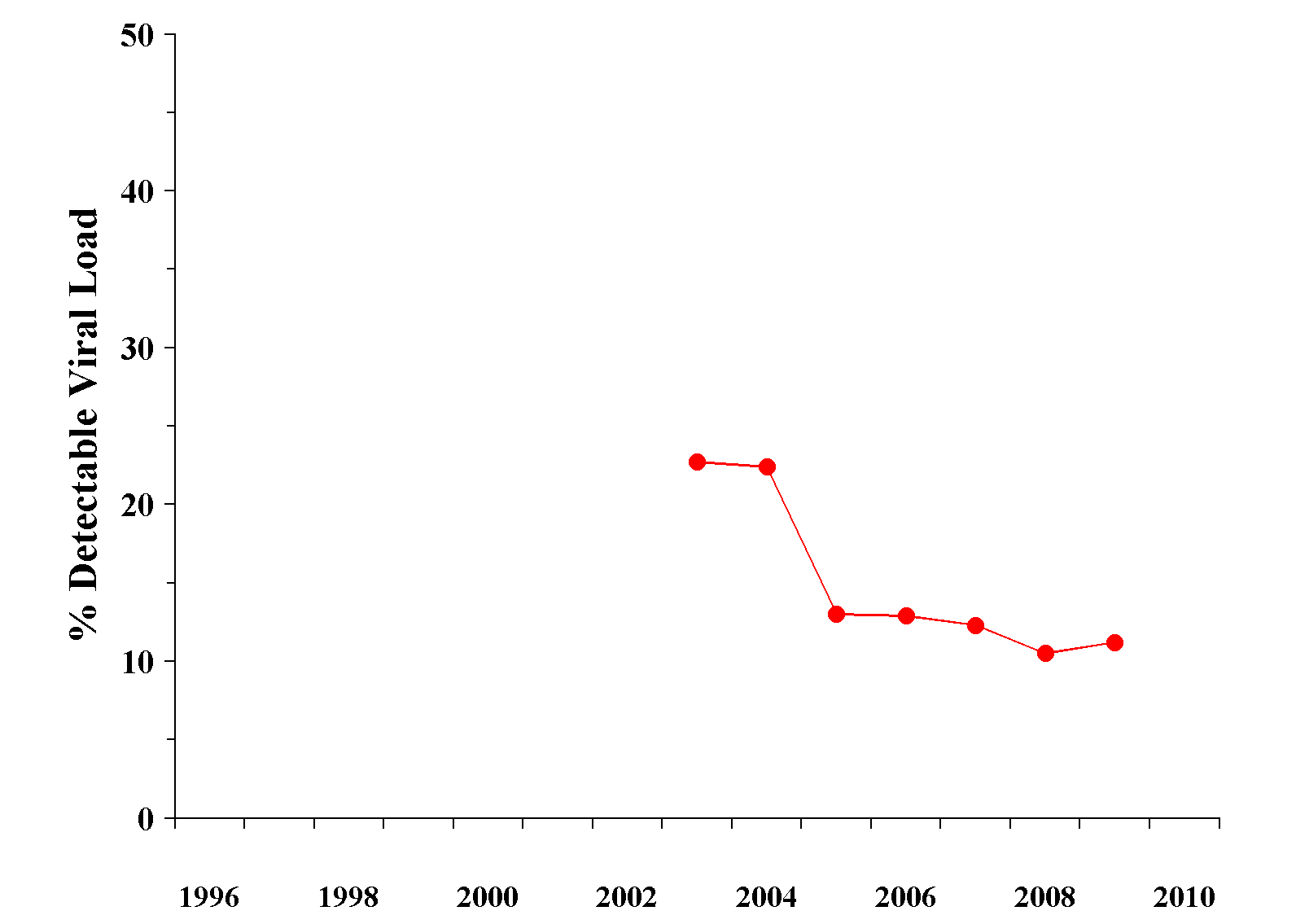  The resulting level of treatment failure at any one time in the model population is shown in Figure S1(e). | | | |

**References**

1. Zablotska I, Prestage G, Frankland A, Crawford J, Sutherland R, et al. (2007) Sydney Gay Community Periodic Survey: February 1996 to August 2006. National Centre in HIV Social Research and the National Centre in HIV Epidemiology and Clinical Research, University of New South Wales, Sydney, NSW, Australia.

2. Prestage G, Ferris J, Grierson J, Thorpe R, Zablotska I, et al. (2008) Homosexual men in Australia: population, distribution and HIV prevalence. Sexual Health 5: 97-102.

3. Fogarty A, Mao L, Zablotska I, Salter M, Prestage G, et al. (2006) The Health in Men and Positive Health cohorts: A comparison of trends in the health and sexual behaviour of HIV-negative and HIV-positive gay men, 2002–2005. National Centre in HIV Social Research, University of New South Wales, Sydney, NSW, Australia.

4. Prestage GP, Hudson J, Down I, Bradley J, Corrigan N, et al. (2009) Gay Men Who Engage in Group Sex are at Increased Risk of HIV Infection and Onward Transmission. AIDS and behav 13: 724-730

5. Colfax GN, Buchbinder SP, Cornelisse PG, Vittinghoff E, Mayer K, et al. (2002) Sexual risk behaviors and implications for secondary HIV transmission during and after HIV seroconversion. AIDS 16: 1529-1535.

6. Marks G, Crepaz N, Senterfitt JW, Janssen RS (2005) Meta-analysis of high-risk sexual behavior in persons aware and unaware they are infected with HIV in the United States: implications for HIV prevention programs. JAIDS 39: 446-453.

7. Valleroy La (2000) HIV Prevalence and Associated Risks in Young Men Who Have Sex With Men. JAMA 284: 198-204.

8. Wilson DP (2009) Modelling based on Australian HIV notifications data suggests homosexual age mixing is primarily assortative. JAIDS 51: 356-360.

9. Quinn TC, Wawer MJ, Sewankambo N, Serwadda D, Li C, et al. (2000) Viral Load and Heterosexual Transmission of Human Immunodeficiency Virus Type 1. New Engl J Med 342: 921.

10. Attia S, Egger M, Müller M, Zwahlen M, Low N (2009) Sexual transmission of HIV according to viral load and antiretroviral therapy: systematic review and meta-analysis. AIDS 23: 1397-1404.

11. Jin F, Jansson J, Law M, Prestage GP, Zablotska I, et al. (2010) Per-contact probability of HIV transmission in homosexual men in Sydney in the era of HAART. AIDS 24: 907-913.

12. Zablotska IB, Imrie J, Bourne C, Grulich AE, Frankland A, et al. (2008) Improvements in sexual health testing among gay men in Sydney, Australia, 2003–2007. Int J STD AIDS 19: 758-760.

13. Templeton DJ, Mao L, Prestage G, Kaldor JM, Kippax S, et al. (2006) Demographic predictors of circumcision status in a community-based sample of homosexual men in Sydney, Australia. SexHealth 3: 191.

14. Australian College of Paediatrics (1996) Position Statement: Routine Circumcision of Normal Male Infants and Boys. Parkville, Victoria.

15. Xu B, Goldman H (2008) Newborn circumcision in Victoria, Australia: reasons and parental attitudes. ANZ J Surg 78: 1019-1022.

16. Wirth JL (1986) Circumcision in Australia: an update. Aust Paediatr J 22: 225-226.

17. Spilsbury K, Semmens JB, Wisniewski ZS, Holman CDAJ (2003) Routine circumcision practice in Western Australia 1981-1999. ANZ ANZ J Surg 73: 610-614.

18. Van de Ven P, Mao L, Fogarty A, Rawstorne P, Crawford J, et al. (2005) Undetectable viral load is associated with sexual risk taking in HIV serodiscordant gay couples in Sydney. AIDS 19: 179-184.

19. Jin F, Crawford J, Prestage GP, Zablotska I, Imrie J, et al. (2009) Unprotected anal intercourse, risk reduction behaviours, and subsequent HIV infection in a cohort of homosexual men. AIDS 23: 243-252.

20. Jin F, Prestage GP, Mao L, Kippax SC, Pell CM, et al. (2006) Transmission of herpes simplex virus types 1 and 2 in a prospective cohort of HIV-negative gay men: the health in men study. J Infect Dis 194: 561-570.

21. Wilson DP, Regan DG, Heymer K-J, Jin F, Prestage GP, et al. (2010) Serosorting may increase the risk of HIV acquisition among men who have sex with men. Sex Transm Dis 37: 13-17.

22. Mellors JW, Muñoz a, Giorgi JV, Margolick JB, Tassoni CJ, et al. (1997) Plasma viral load and CD4+ lymphocytes as prognostic markers of HIV-1 infection. Ann Intern Med 126: 946-954.

23. Mocroft A, Phillips A, Gatell J, Ledergerber B, Fisher M, et al. (2007) Normalisation of CD4 counts in patients with HIV-1 infection and maximum virological suppression who are taking combination antiretroviral therapy: an observational cohort study. Lancet 370: 407-413.

24. Smith C, d'Arminio Monforte A, de Wit S, Friis-Moller N, Lundgren J, et al. (2008) Causes of death in the D:A:D study-initial results.

25. Ledergerber B, Lundgren JD, Walker aS, Sabin C, Justice A, et al. (2004) Predictors of trend in CD4-positive T-cell count and mortality among HIV-1-infected individuals with virological failure to all three antiretroviral-drug classes. Lancet 364: 51-62.

26. Wilson DP, Law MG, Grulich AE, Cooper DA, Kaldor JM (2008) Relation between HIV viral load and infectiousness: a model-based analysis. Lancet 372: 314-320.

27. Smith CJ, Phillips AN, Hill T, Fisher M, Gazzard B, et al. (2005) The rate of viral rebound after attainment of an HIV load <50 copies/mL according to specific antiretroviral drugs in use: results from a multicenter cohort study. J Infect Dis 192: 1387-1397.

28. Weller S, Davis K (2002) Condom effectiveness in reducing heterosexual HIV transmission. Cochrane Db Syst Rev: CD003255.

29. D'Anna L, Margolis A, Warner L, Korosteleva O, O'Donnell L, et al. (2012) Condom use problems during anal sex among men who have sex with men (MSM): Findings from the Safe in the City Study. AIDS Care 24: 1028-1038.

30. Fitch TJ, Stine C, Hagar DW, Mann J, Adam MB, et al. (2002) Condom Effectiveness: Factors that influence risk reduction. Sex Transm Dis 29: 811-817.
